# Supplementary figures and images for: This and that in depression: Cross-linguistic semantic effects
Source: PLOS Ment Health. 2025 Sep 24;2(9):e0000438. doi: 10.1371/journal.pmen.0000438 (PMC12798180; doi:10.1371/journal.pmen.0000438)

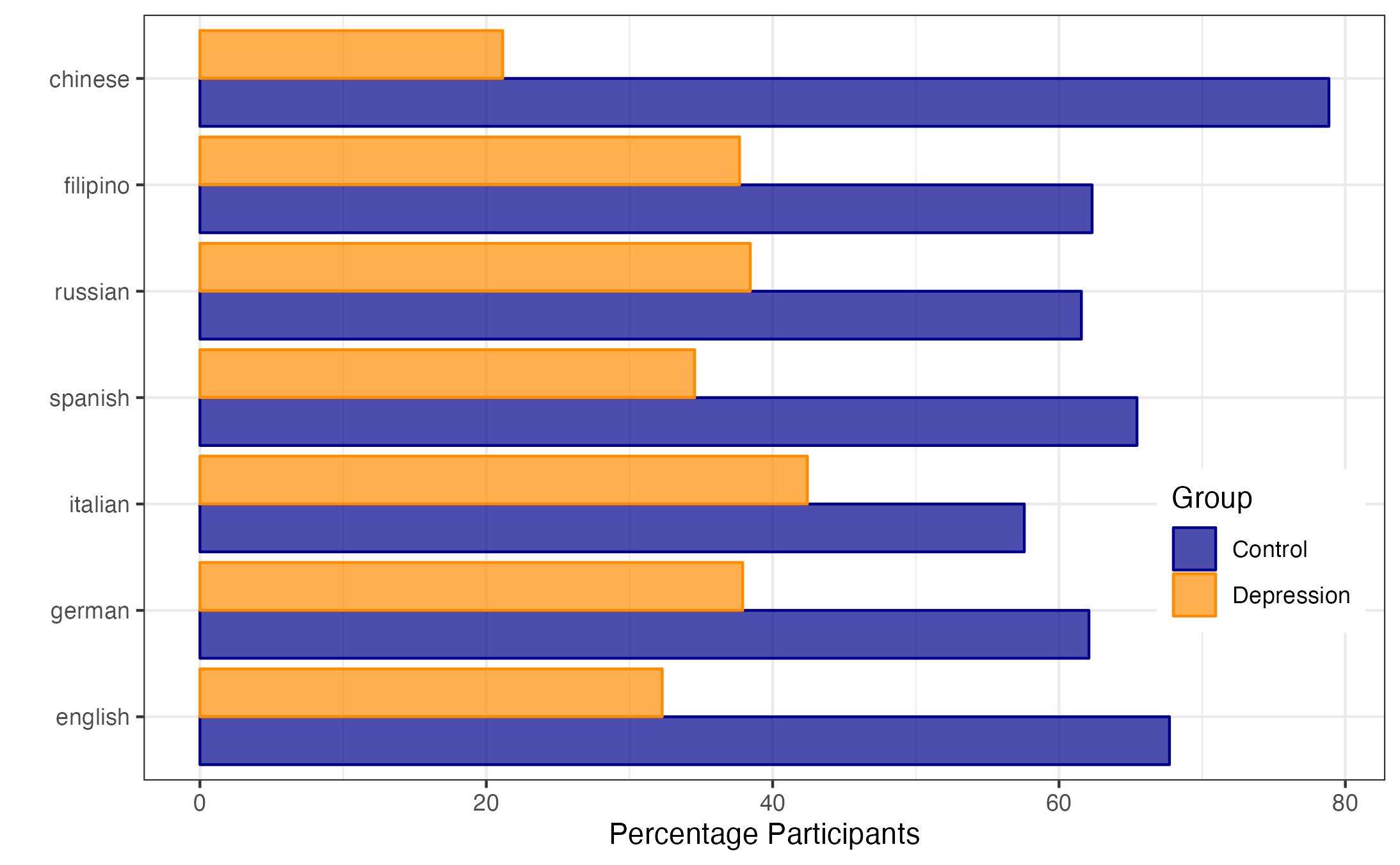

Supplement: S1 Fig — (TIF) [file pmen.0000438.s001.tif]

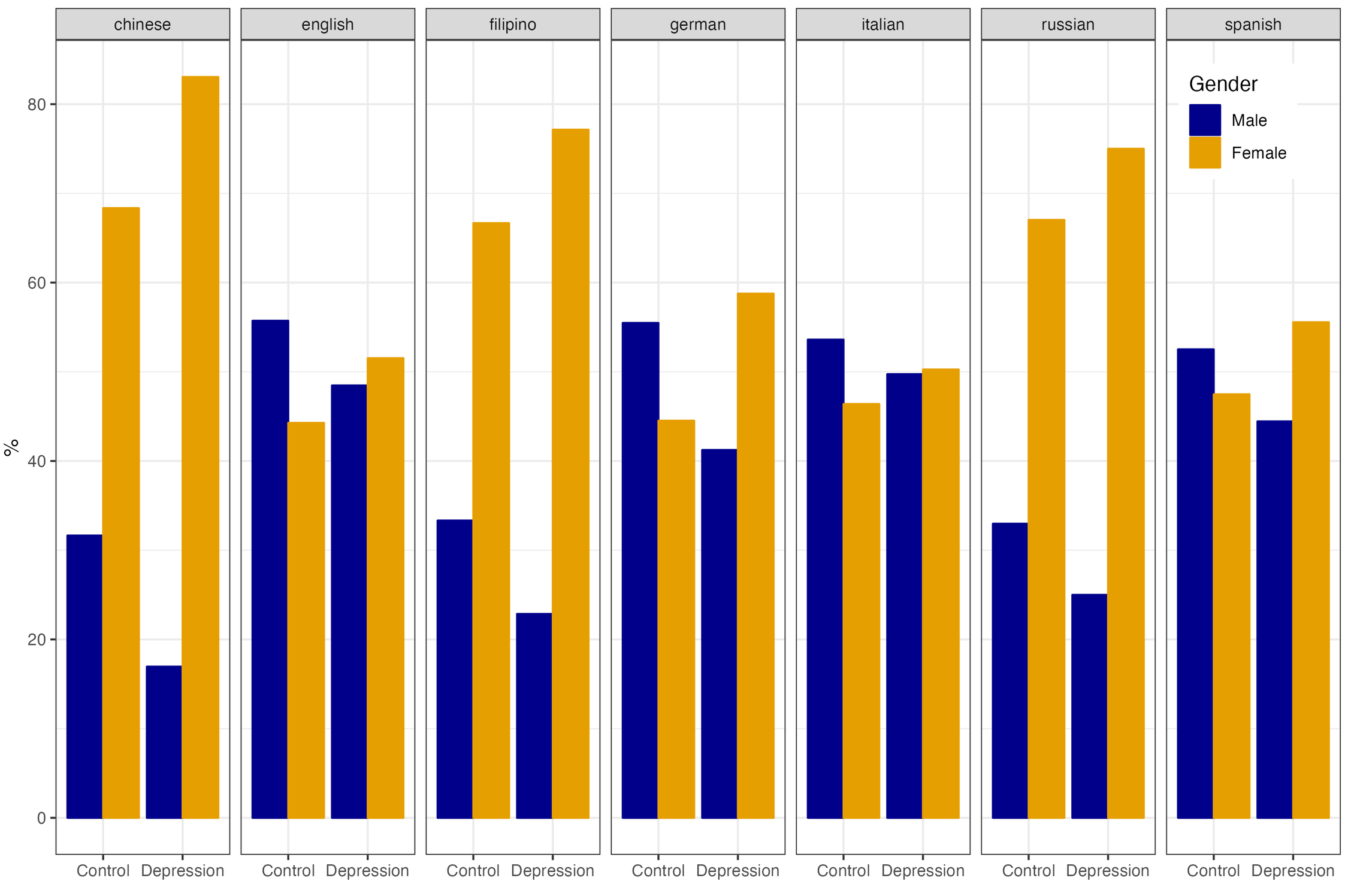

Supplement: S2 Fig — (TIF) [file pmen.0000438.s002.tif]

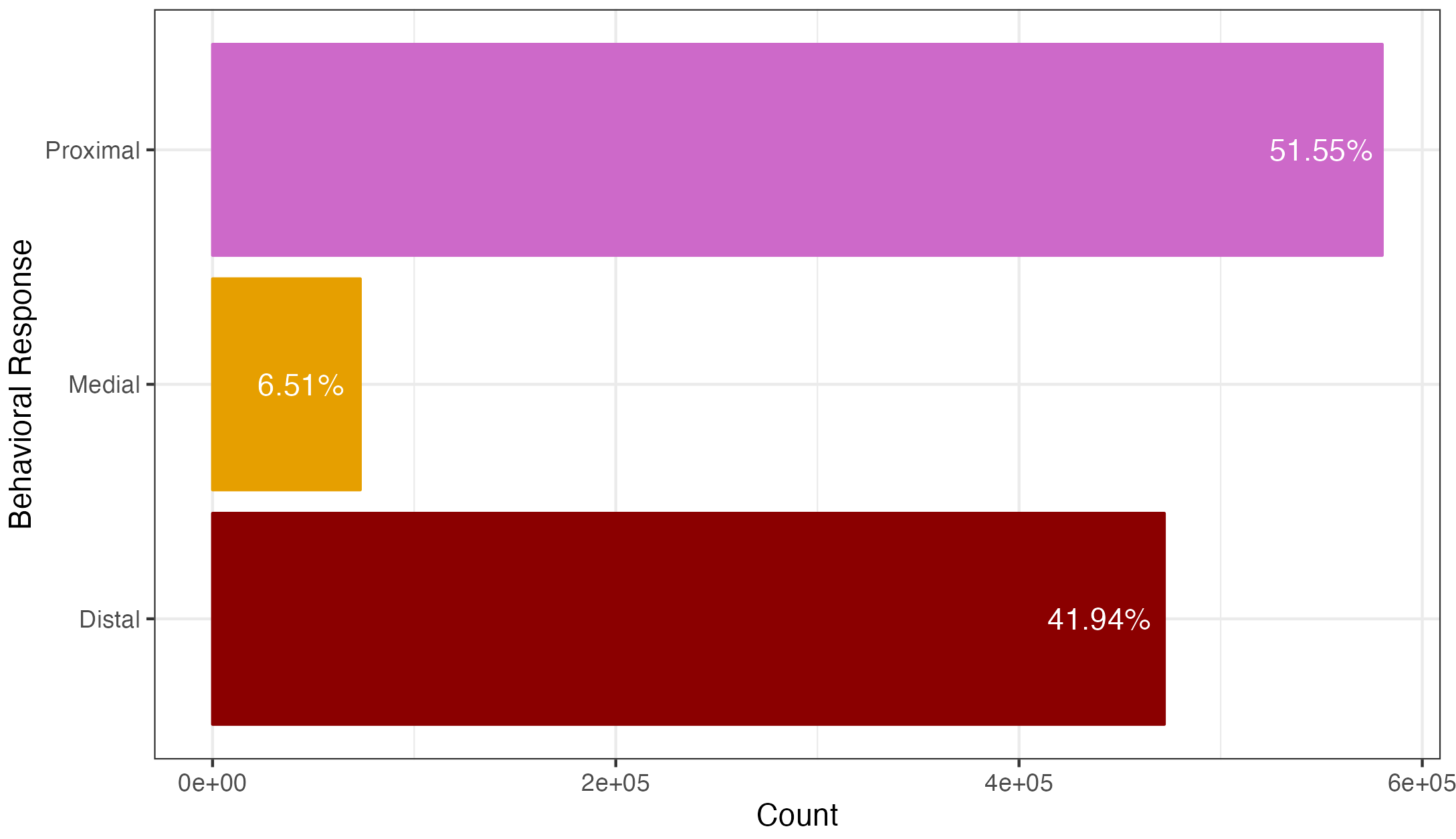

Supplement: S3 Fig — (TIF) [file pmen.0000438.s003.tif]

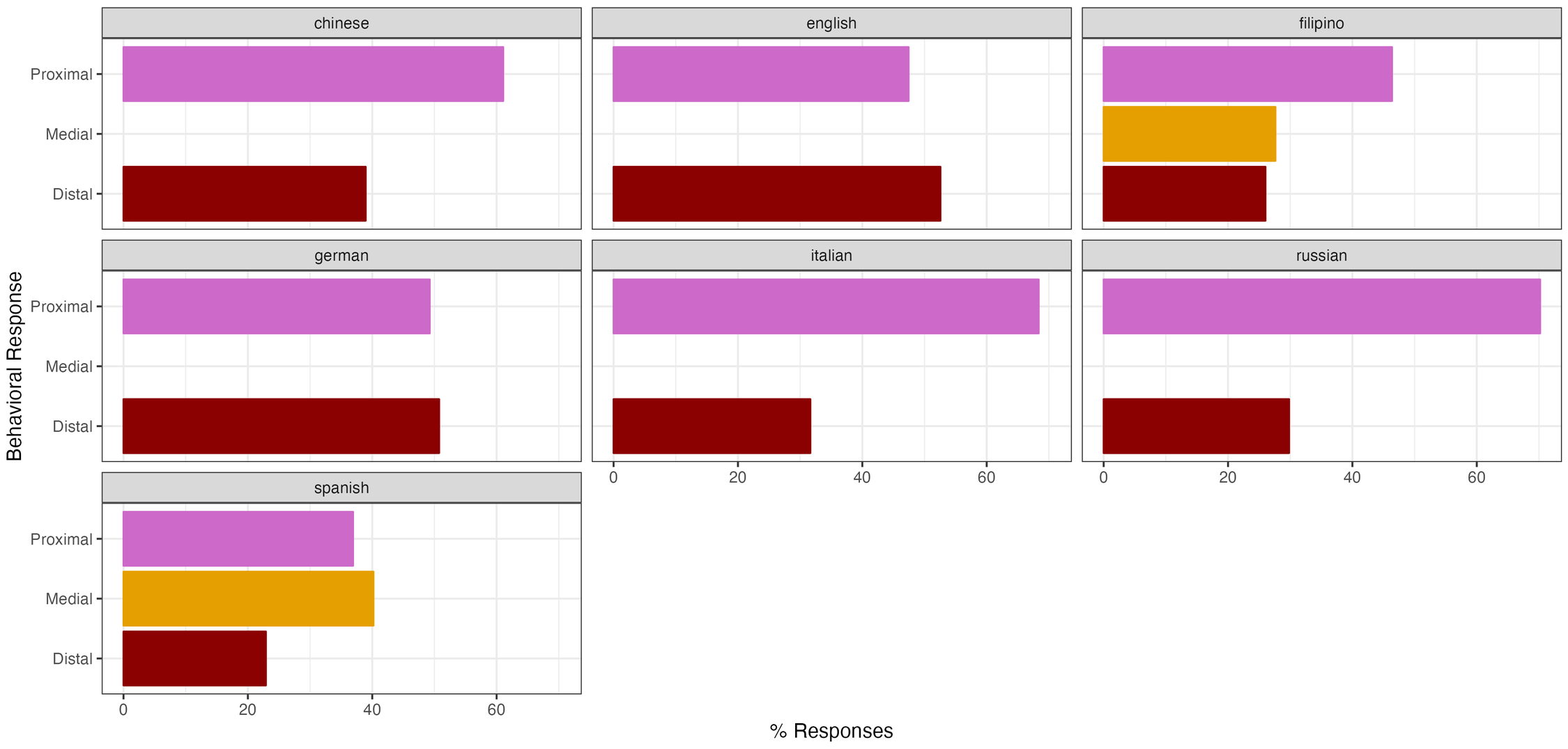

Supplement: S4 Fig — (TIF) [file pmen.0000438.s004.tif]

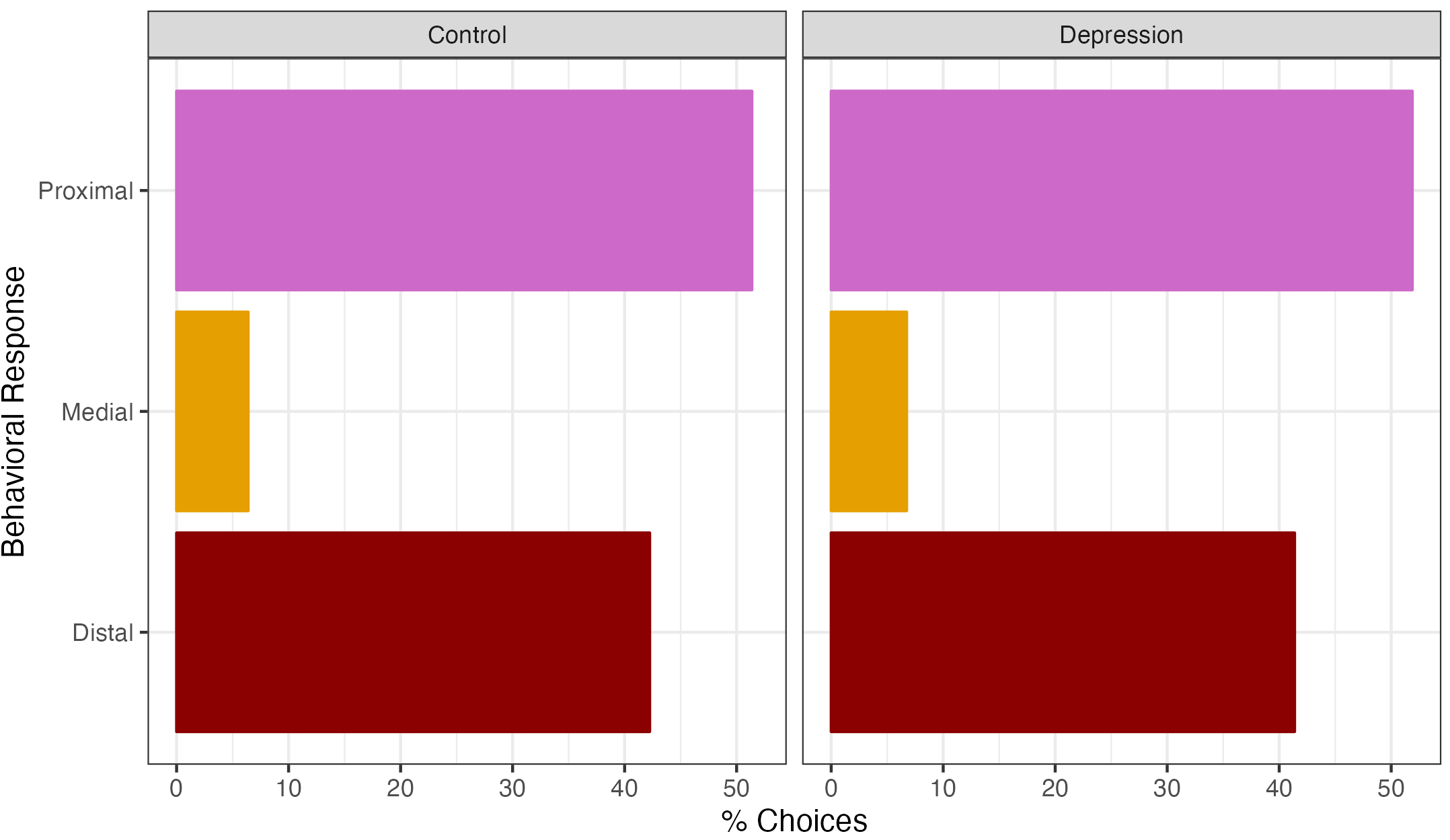

Supplement: S5 Fig — (TIF) [file pmen.0000438.s005.tif]

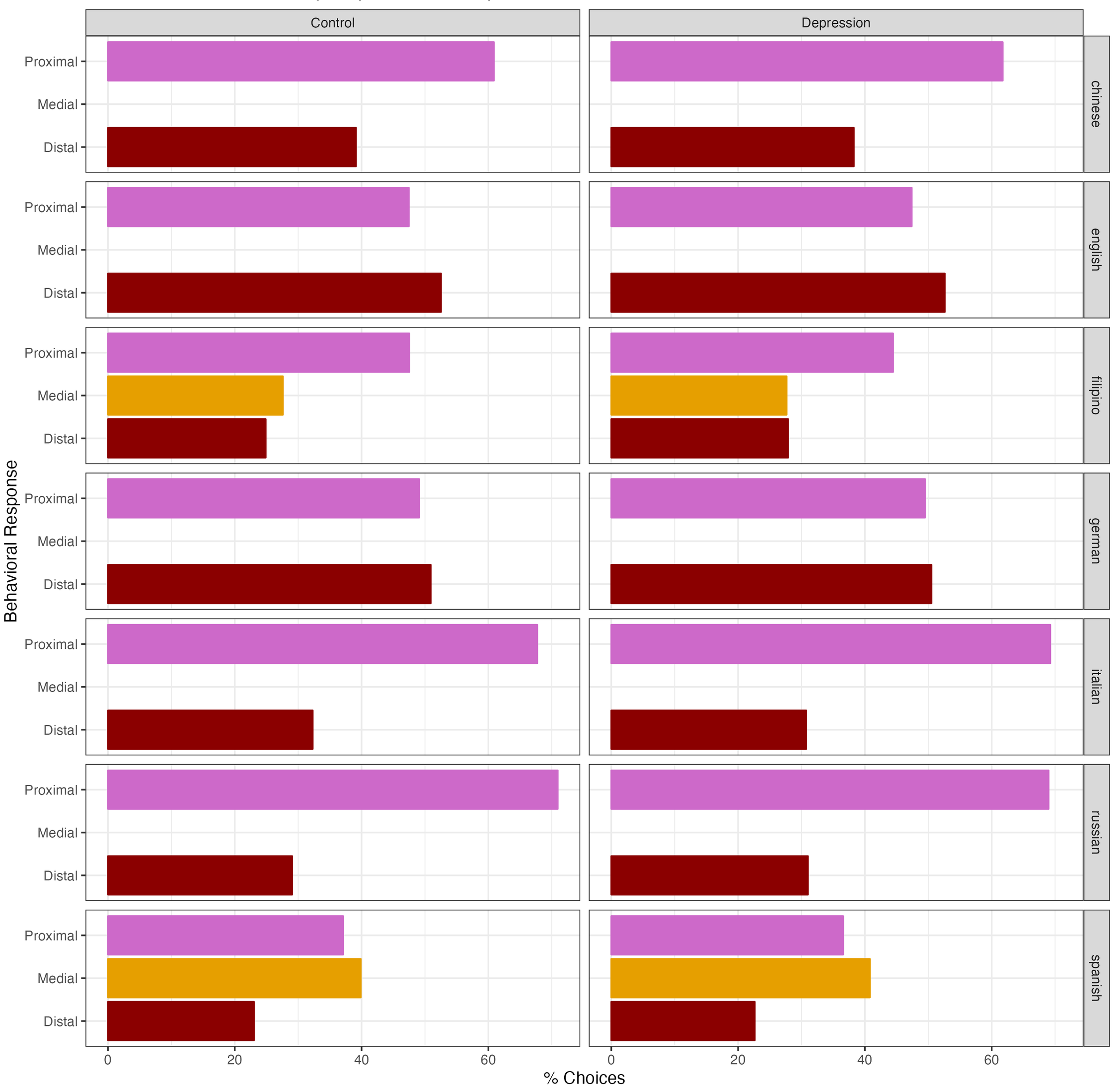

Supplement: S6 Fig — (TIF) [file pmen.0000438.s006.tif]

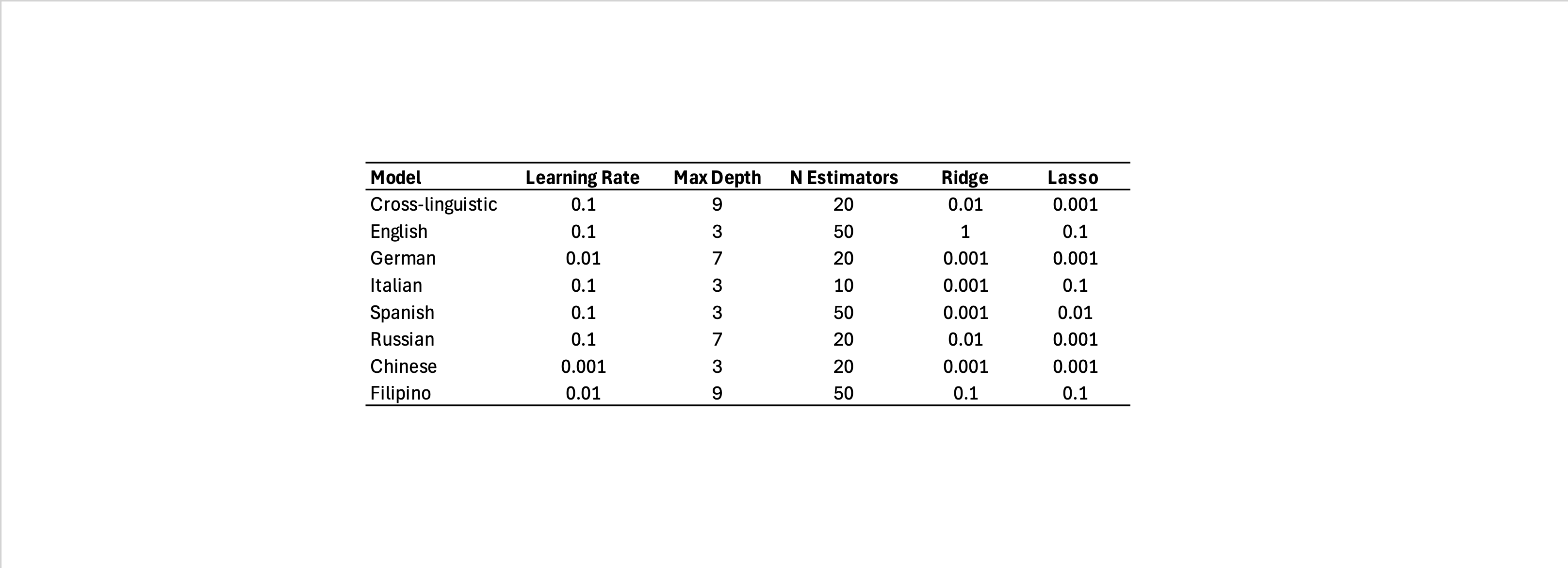

Supplement: S1 Table — (TIFF) [file pmen.0000438.s007.tiff]
