# Supplementary material for: This and that in depression: Cross-linguistic semantic effects
Source: PLOS Ment Health. 2025 Sep 24;2(9):e0000438. doi: 10.1371/journal.pmen.0000438 (PMC12798180; doi:10.1371/journal.pmen.0000438)

S7 Appendix

Figure A. English sample: Semantic score distributions across participants

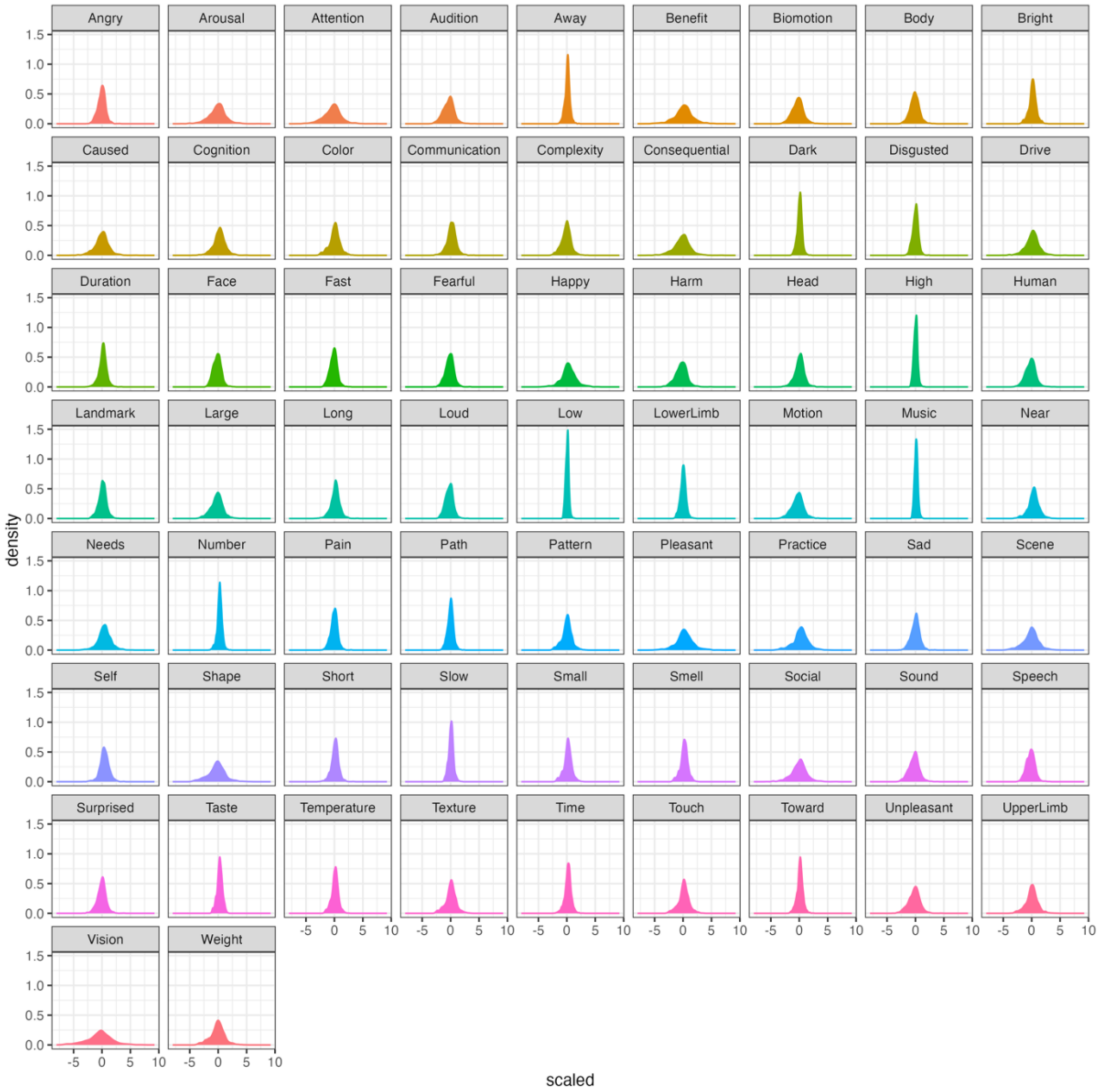

Figure B. German sample: Semantic score distributions across participants

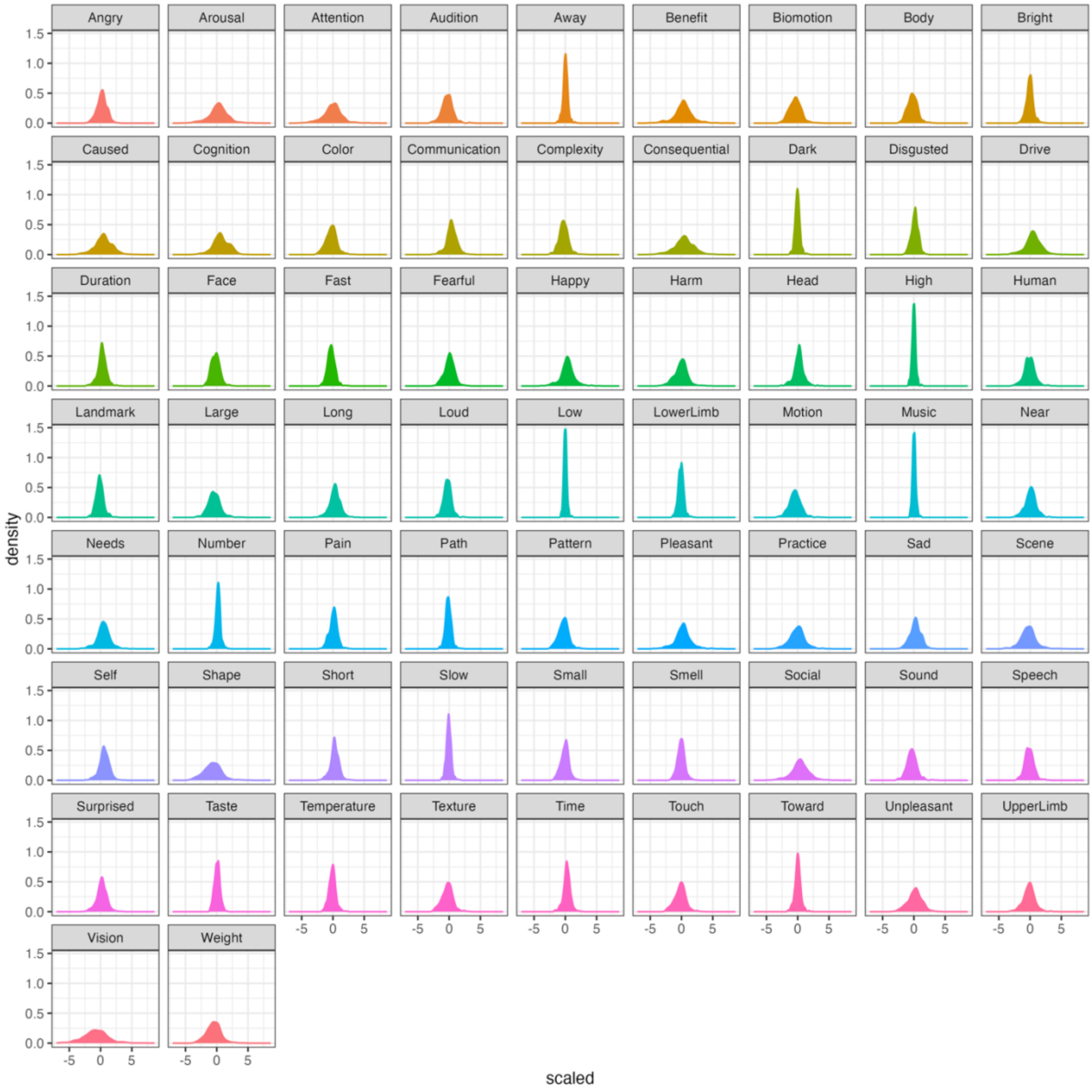

Figure C. Italian sample: Semantic score distributions across participants

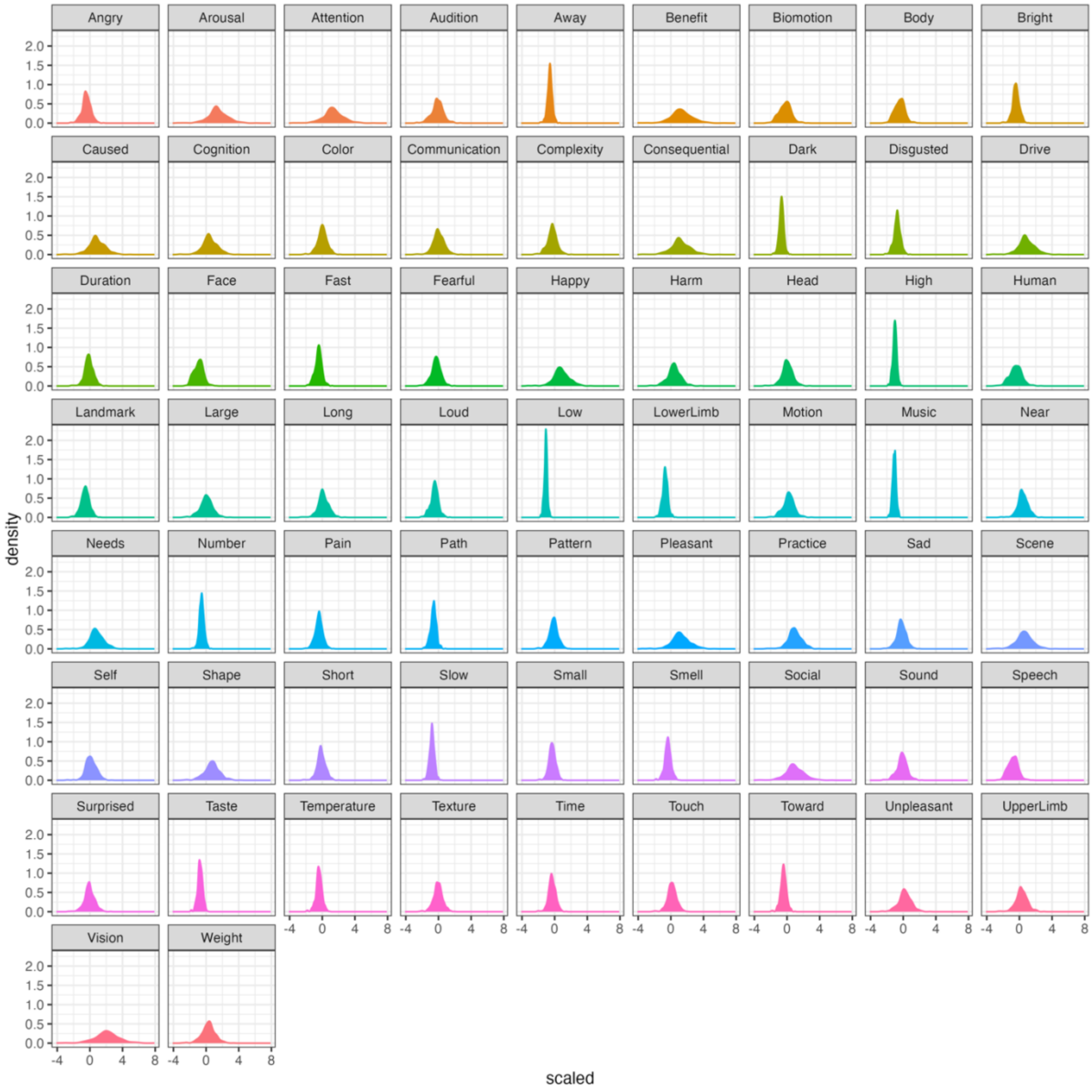

Figure D. Spanish sample: Semantic score distributions across participants

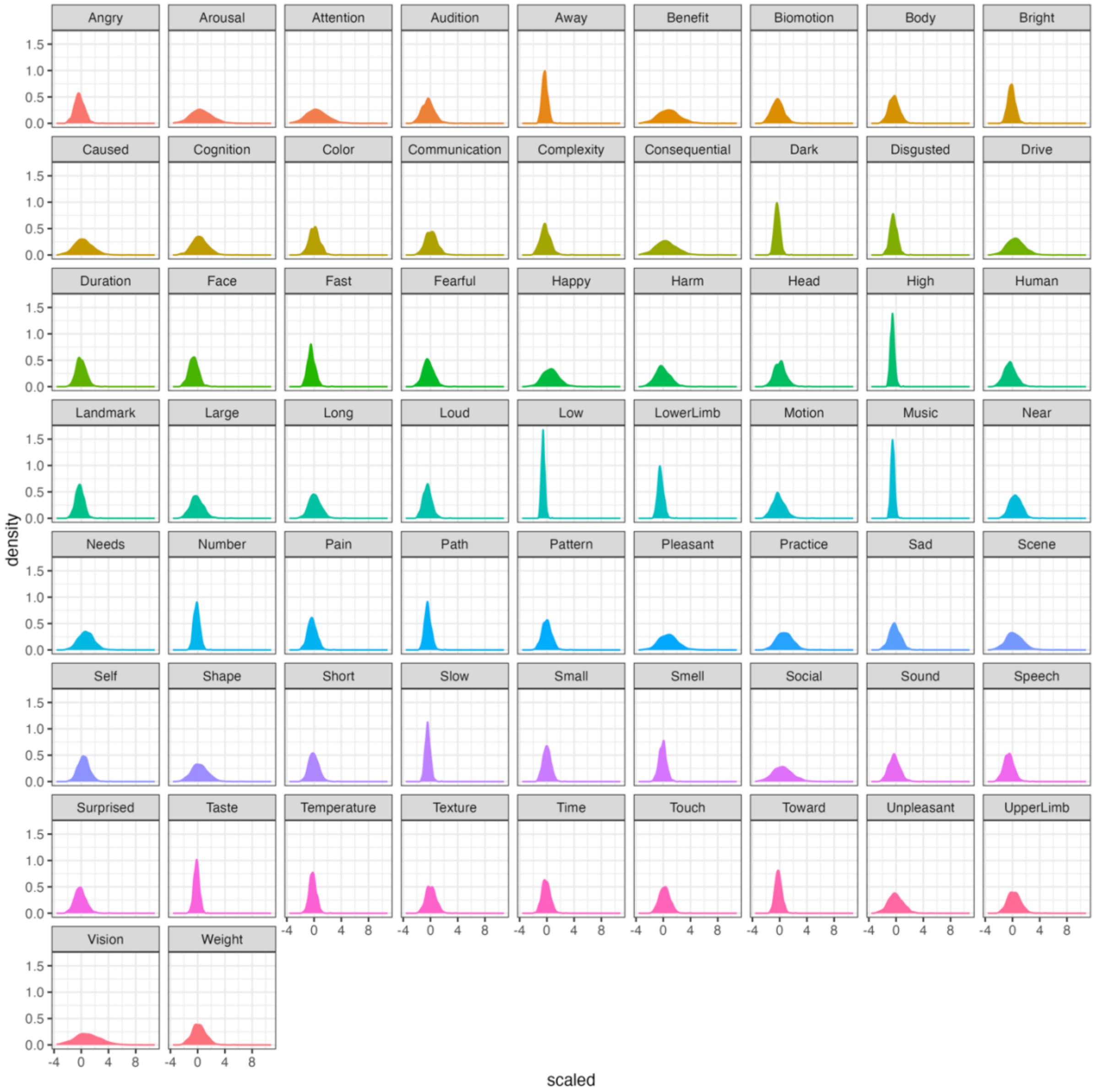

Figure E. Chinese sample: Semantic score distributions across participants

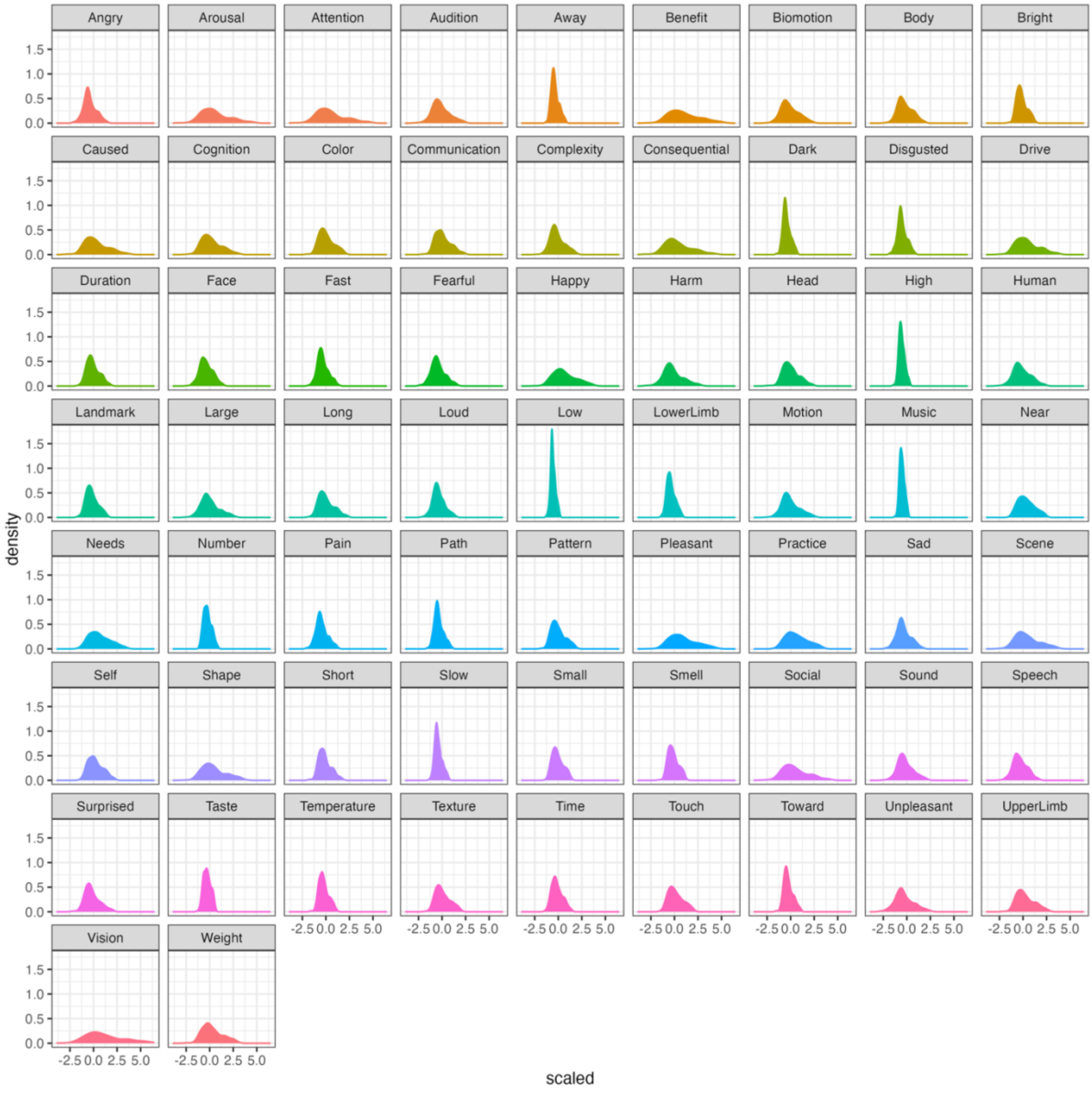

Figure F. Russian sample: Semantic score distributions across participants

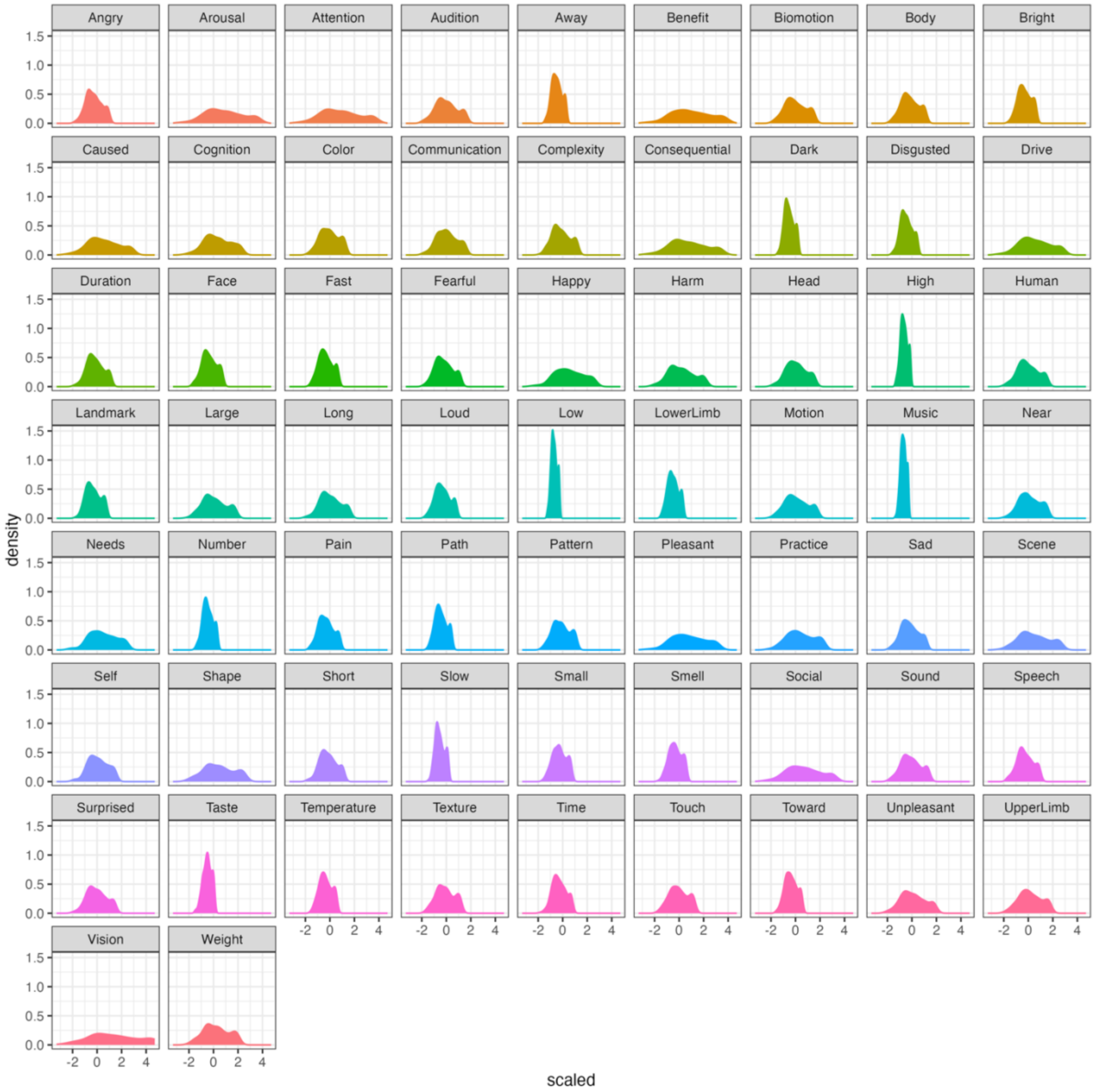

Figure G. Filipino sample: Semantic score distributions across participants

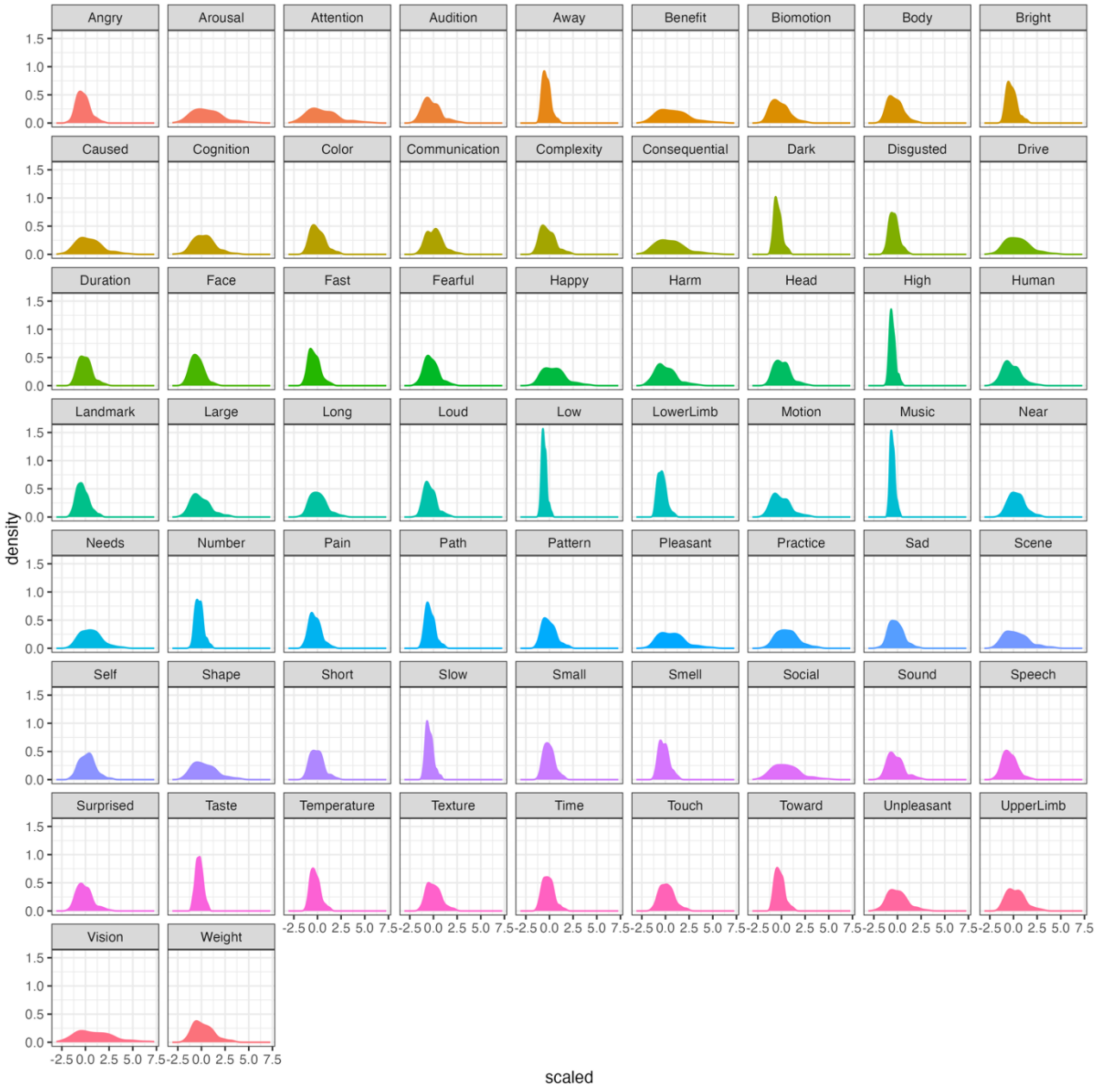

Supplement: S1 Appendix — (PDF) [file pmen.0000438.s008.pdf]
