# Supplementary material for: This and that in depression: Cross-linguistic semantic effects
Source: PLOS Ment Health. 2025 Sep 24;2(9):e0000438. doi: 10.1371/journal.pmen.0000438 (PMC12798180; doi:10.1371/journal.pmen.0000438)

S8 Appendix

Figure A. English sample: Bootstrapped semantic feature importance (SHAP)

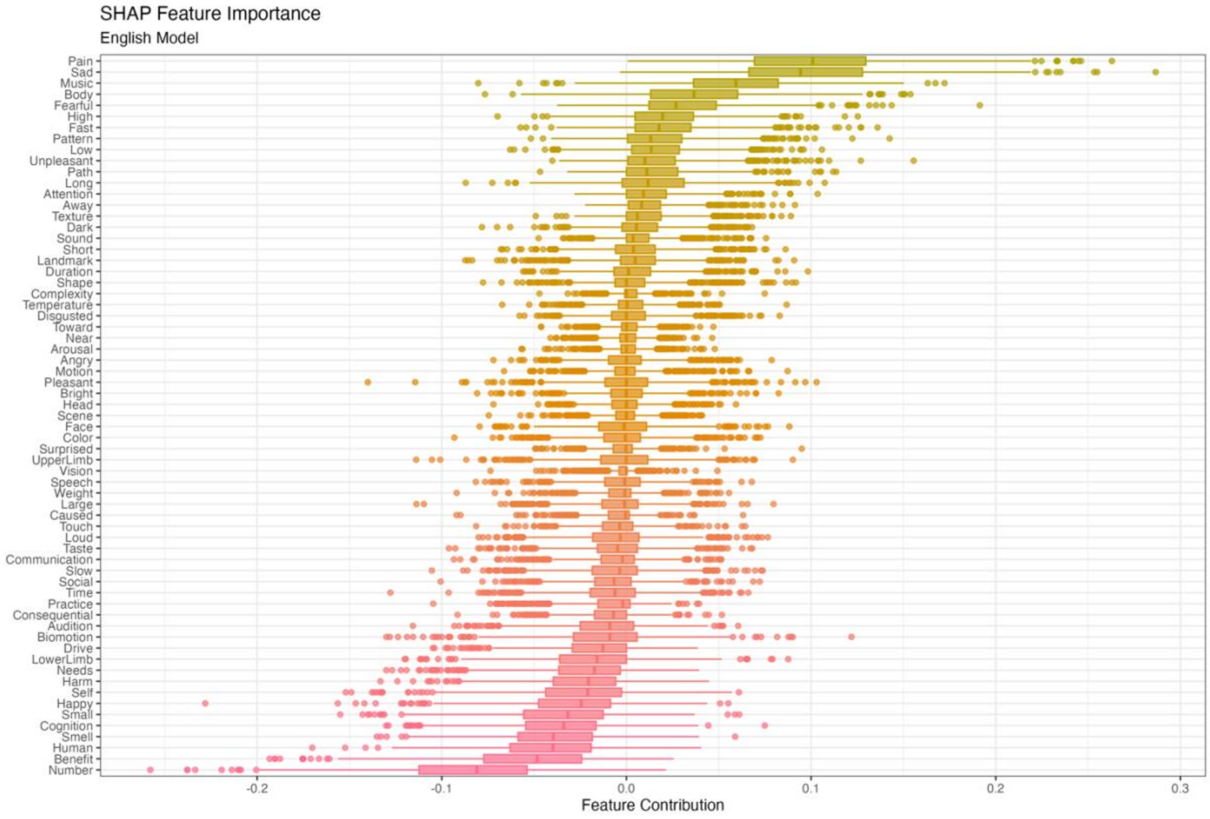

Figure B. German sample: Bootstrapped semantic feature importance (SHAP)

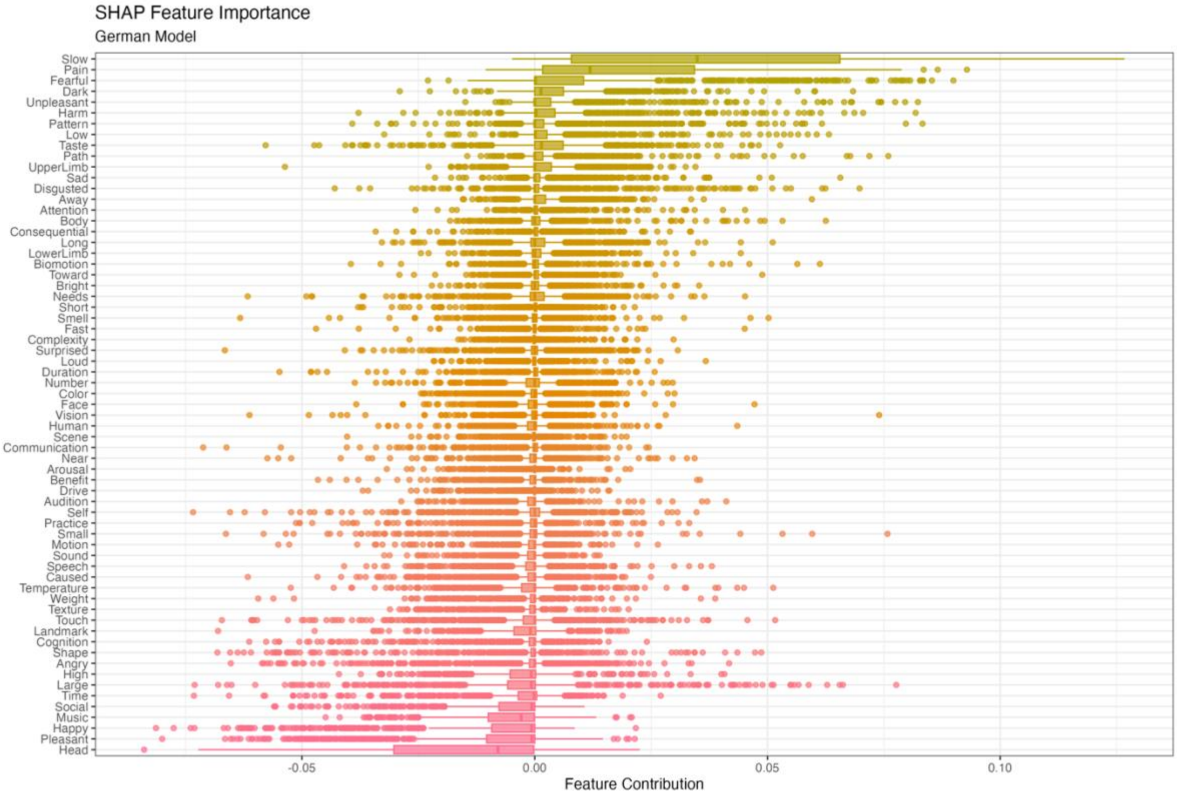

Figure C. Italian sample: Bootstrapped semantic feature importance (SHAP)

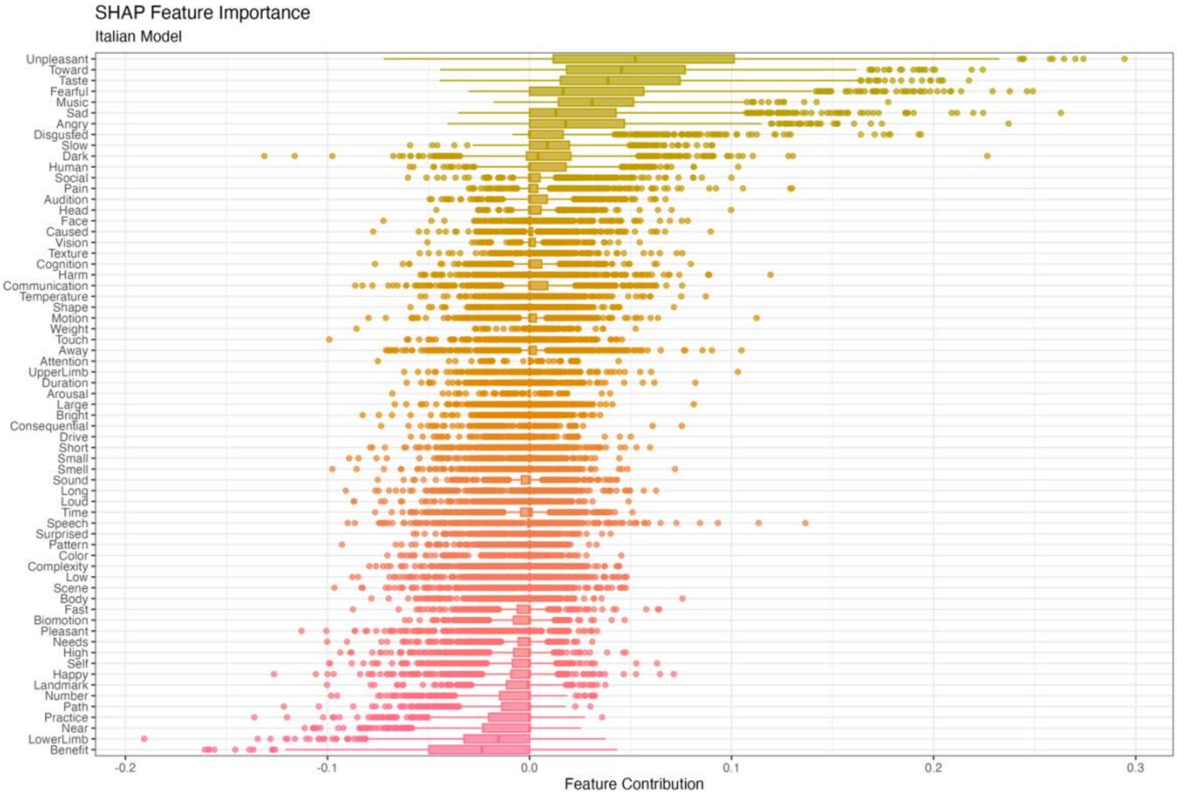

Figure D. Spanish sample: Bootstrapped semantic feature importance (SHAP)

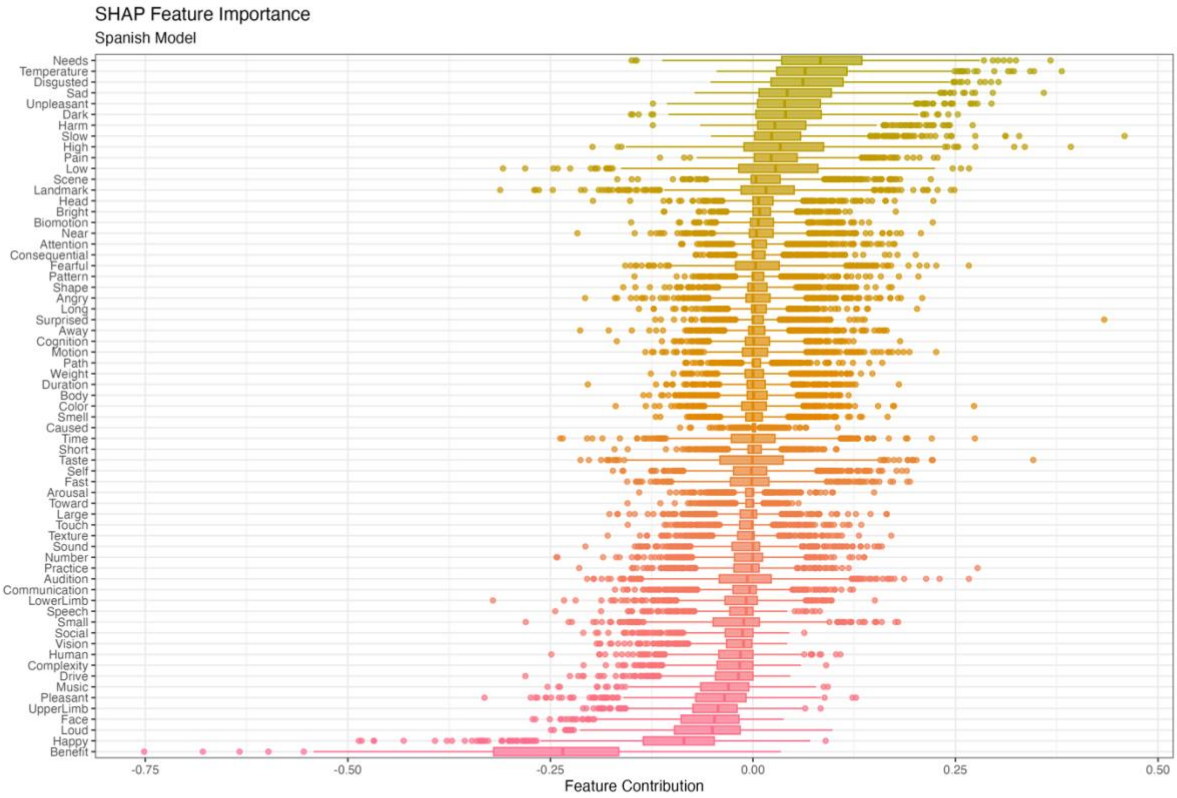

Figure E. Chinese sample: Bootstrapped semantic feature importance (SHAP)

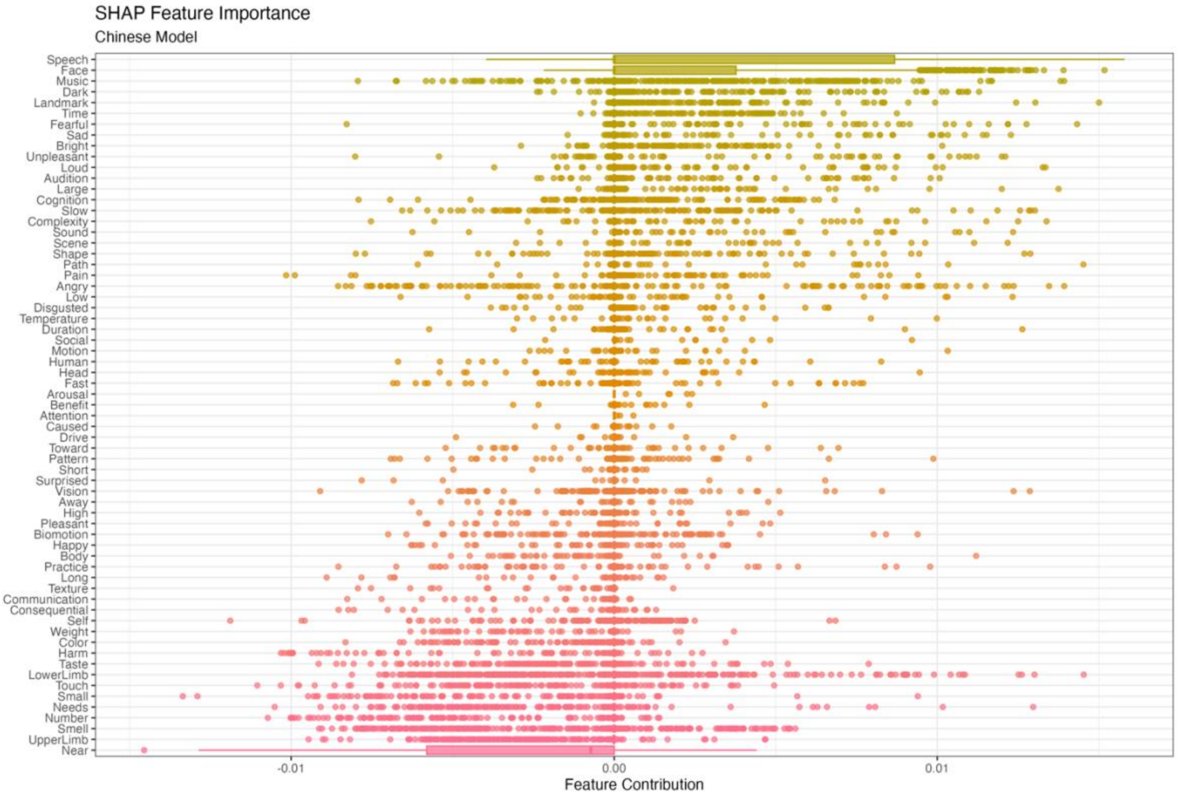

Figure F. Russian sample: Bootstrapped semantic feature importance (SHAP)

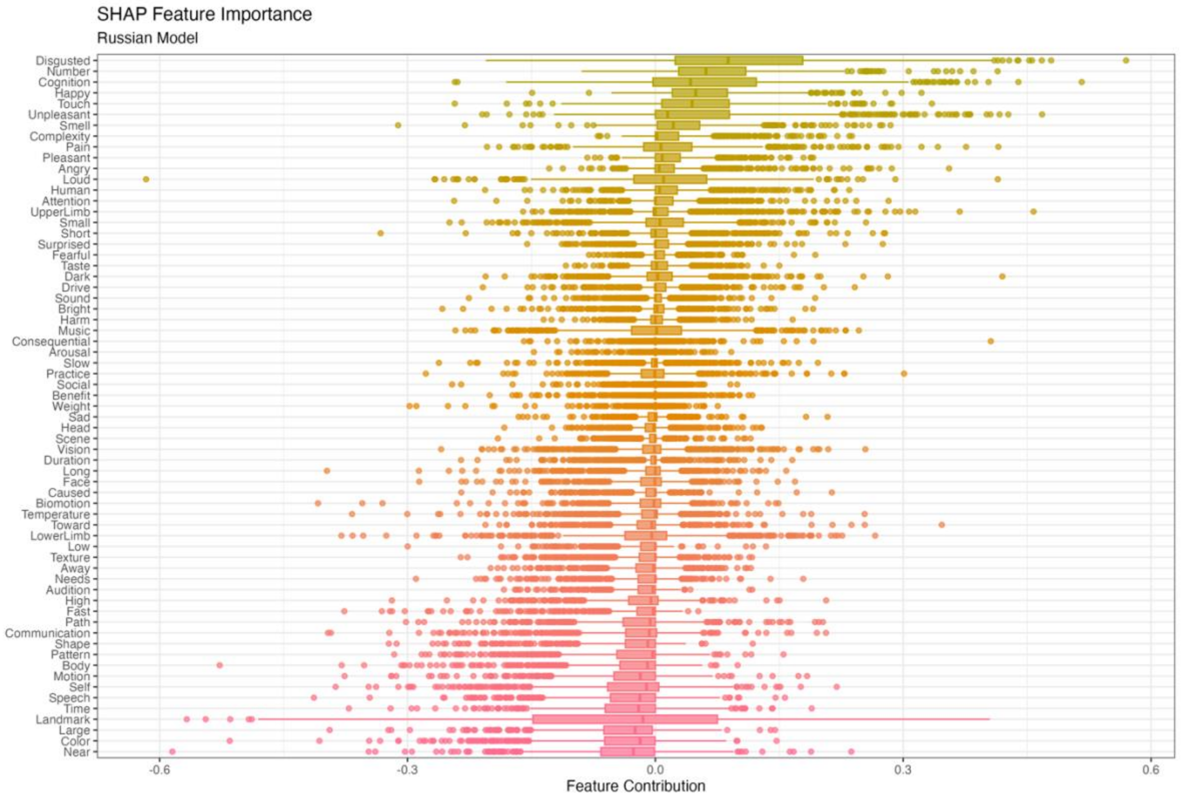

Figure G. Filipino sample: Bootstrapped semantic feature importance (SHAP)

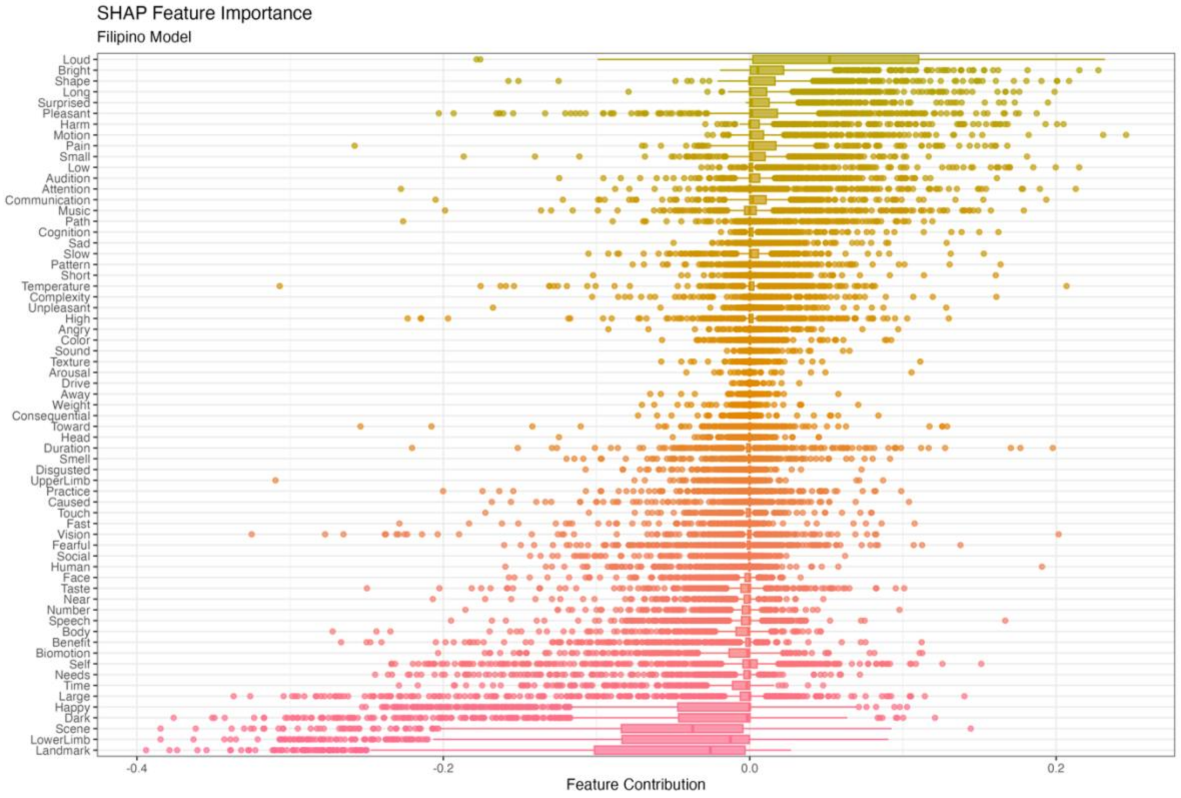

Supplement: S2 Appendix — (PDF) [file pmen.0000438.s009.pdf]
