# Supplementary material for: This and that in depression: Cross-linguistic semantic effects
Source: PLOS Ment Health. 2025 Sep 24;2(9):e0000438. doi: 10.1371/journal.pmen.0000438 (PMC12798180; doi:10.1371/journal.pmen.0000438)
Supplement: S1 Supplementary Experimental Procedures — (PDF) [file pmen.0000438.s010.pdf]

S1 Supplementary Experimental Procedures

1 Notes on translations of the DCT

The German, Spanish, and Italian translations of the DCT paradigm were conducted by authors of the paper who are native speakers of the respective languages; the German translations was done by CV; the Italian translation by RR; the Spanish translation by ET. The Russian translation by a native speaker. For these four languages, the first author initially used machine translation to translate all nouns, which were subsequently validated and corrected by the native speaker in two stages; 1) validation and correction of the translation of the raw word list and corresponding demonstratives, including spelling, grammar, double meanings, etc., and 2) walk-through of the full experiment to check that each individual trial was correct, sensible, and comprehensible. The Chinese translation was initially performed by author PTW in collaboration with a native Chinese speaker. The Tagalog Filipino translation was done by native speaker. For these two languages, after initial translation the native speakers similarly completed a full walk-through of each individual trial and made corrections until a fully correct and comprehensible translation was ensured.

All translators were carefully instructed and informed about the purpose and structure of the task and all completed the English version of the DCT prior to completing the translations, to familiarize themselves with the task format. Translators were instructed to translate all items as nouns. During the translation process, all translators made notes on individual task items (words) for which multiple translations with different meanings could be made or other discrepancies of the like. These were all monitored and discussed with the first author, who ensured that such discrepancies were translated to maximize alignment between languages as much as possible (e.g., the English word "party" could be translated either in the sense of a celebratory event or a political party. In this case, the first author instructed all translators to translate it in the former sense). English words which take different forms in other languages depending on the gender (e.g., "journalist" which in Spanish and Italian acquires different endings for males and females), were translated and included in the task with both possible endings ("reportero/a").

Task instructions preceding the experiment were fully translated by all native speakers of each respective language.

Publicly available versions of the PHQ-9 scale assessing depression symptom severity were used for all languages. They are found here:
German version PHQ-9
Italian version PHQ-9
Spanish version PHQ-9
Chinese version PHQ-9
Russian version PHQ-9
Tagalog Filipino version PHQ-9

2 Notes on demonstrative systems

German has two demonstratives, "hier" and "da", corresponding to a proximal versus distal distinction. These terms are demonstrative *adverbs* (translating to the English "here" and "there"), and must be preceded by the definite noun form indicated by one of the three gender-specific definite articles ("der"/"die"/"das"). In natural speech the demonstrative would follow the definite noun phrase ("die Familie hier" = "this family"). In the German translation of the DCT, trials were presented as [def.articl. + noun] under which the response options ["hier" / "da"] were presented.

Italian has two demonstratives, corresponding to a proximal (“questo”) and distal (“quello”) distinction. These are demonstrative adjectives, similar to English, and changes form according to the gender and first letter of the noun. Proximal demonstratives used in the DCT included “quest’”, “questo”, “questa”, “questo/a”, “questi”, “quel”, “questi” and distal demonstratives included “quell’”, “quello”, “quella”, “quello/a”, “quelli”, “quel/la”, “quei”. Demonstratives including both masculine and feminine forms (“questo/a”, “quello/a”, “quel/la”) were used for nouns that can be either feminine or masculine (e.g. “paziente”, “patient” in English). In natural speech demonstratives typically precedes the noun (“questa famiglia” = “this family”). In the Italian version of the DCT, trials were presented as [noun] under which the respective two demonstratives were presented.

Spanish has three demonstratives corresponding to a proximal (“esta”), medial (“esa”) and distal (“aquella”) form. These function as determiners and changes according to the gender of the word. As for Italian, some of the DCT nouns takes a plural form in Spanish, for which the plural demonstrative forms were used. Proximal demonstratives used in the Spanish DCT included “esta”, “este”, “este/a”, “estos”, and “estas”, the medial demonstratives included “esa”, “ese”, “ese/a”, “esos”, and “esas”, and the distal demonstratives included “aquella”, “aquel”, “aquel/lla”, “aquellos”, and “aquellas”. Demonstratives including both masculine and feminine forms (“este/a”, “ese/a”, “aquel/la”) were used for nouns that can be either feminine or masculine (e.g. “paciente”, “patient” in English). In natural speech, demonstratives typically precedes the noun (“esta familia” = “this family”). In the Spanish version of the DCT, trials were presented as [noun] under which the respective three demonstratives were presented.

Tagalog Filipino has three demonstratives corresponding to a proximal (“ito”), medial (“iyan”) and distal (“iyon”) form. Demonstrative reference in Tagalog requires both the demonstrative form, the preceding “ang” marking absolutive case, and the addition of either an “-ng” affix to the noun or the the linker “na” preceding the demonstrative, depending on the spelling of the noun (e.g., for the noun “world”, “mundo” becomes “mundong”, “this world” translates to “ang mundong ito”, “this life” translates to “ang buhay na ito”). In the Tagalog Filipino version of the DCT, nouns were presented as [abs. marker + noun + affix/linker] below which the three demonstratives were presented [“ito” / “iyan” / “iyon”].

Russian has two demonstratives corresponding to a proximal (этот) and distal (тот) forms. They function as determiners and change according to the gender of the noun. Some of the DCT nouns take a plural form in Russian, for which the plural demonstrative forms were used. Proximal demonstratives used in the Russian DCT include эта, этот, это, эти, and the distal demonstratives included та, тот, то, те. In natural speech, the demonstrative precedes the noun (эта семья = "this family"). In the Russian version of the DCT, trials were presented as [noun], under which the respective two demonstratives were presented.

Mandarin Chinese has two demonstratives corresponding to a proximal (这, zhè) and distal (那, nà) form. They function as determiners and take the same form for all nouns, as in the English case. However, Chinese nouns require a measure word indicating the "unit"of the noun and in demonstrative reference the demonstratives are combined with the measure words. The most basic and neutral of these is "个"(gè) translating into "individual"or "unit". Some nouns however, are usually used with more specific measure words, in which case those were used in the given trials. For example, 次 (cì) is used for action nouns (e.g., "riot"), and translates most closely to "times". In natural speech, demonstrative reference typically has the structure [demonstrative] + [measure word] + [noun] (这个家庭 = "this (unit) family"). In the Chinese version of the DCT, nouns were presented as [noun] below which response options were presented as [demonstrative form + measure word].
