# Supplementary material for: This and that in depression: Cross-linguistic semantic effects
Source: PLOS Ment Health. 2025 Sep 24;2(9):e0000438. doi: 10.1371/journal.pmen.0000438 (PMC12798180; doi:10.1371/journal.pmen.0000438)
Supplement: S2 Supplementary Experimental Procedures — (PDF) [file pmen.0000438.s011.pdf]

S2 Supplementary Experimental Procedures

1 Replication analysis: Depression classification from principal component (PC) representations of DCT responses

All analyses were conducted in RStudio, version 4.1.1 Team, 2020.

1.1 Principal Component Analysis (PCA)

The 290 response features of the DCT were subjected to principal component analysis (PCA) to reduce dimensionality and correlation of the input features in subsequent classification models. PCA was conducted with the *stats* R package (Team, 2020) which performs singular value decomposition and returns two matrices; an $m\ x\ d$ matrix with rotation weights of the m original variables on the d principal components (PCs), and an $n\ x\ d$ matrix with scores of the n subjects on the d PCs, calculated as the true data matrix multiplied by the rotation matrix. The subject-level PC scores were inputted as predictors in subsequent classification models.

The first 4 PCs explained most of the variance in the 290 DCT variables, respectively explaining 0.1 (SD=4.7), 0.04 (SD=3.2), 0.03 (SD=2.1), and 0.02 (SD=2.1) proportions of the variance. The remaining components explained less than 1% of the total variance and had SDs below 1.6. The cumulative proportion of variance explained by the first 4 PCs was 19%, and the first 100 PCs explained 60% of the variance.

1.2 Classification models

Two logistic regression models were estimated, classifying outcome group (control vs. depression) based on principal component (PC) representations of the DCT responses. The first model (mDCT) included only DCT responses. The second model (mDCT+GenderAge) included, in addition to DCT behavior, *gender* and *age* as predictors. These demographic features have shown to be associated with language usage (Sap et al., 2014; Schwartz et al., 2013) as well as correlated with depression prevalence (Kupfer, Frank, & Phillips, 2012), and the second model addressed whether accounting for these variables could improve model performance. Performance of the DCT classifiers were compared to two baseline models. One including only *gender* and *age* as predictors of outcome group (mGenderAge), and one random baseline trained to classify a randomly shuffled version of the outcome group from DCT responses (mRandomBaseline).

Prior to model estimation, the data was down-sampled by random seed to balance the prevalence of each outcome class, and subsequently partitioned into train- (=70%) and test sets (=30%) stratified by outcome group. This yielded a training set of n=1124 and a test set of n=482. Model training was performed with the *caret* R package (Kuhn, 2008) using the *glm* method with k=3 repeated 10-fold cross-validation. For the three DCT-based models (mDCT, mDCT+GenderAge and mRandomBaseline), 100 models were trianed and evaluated, iteratively adding a PC as predictor, starting from 1 to the first 100 PCs. This allowed model estimation procedure to identify the optimal number of PCs for the classification task. The model yielding best out-of-sample was identified and reported.

Model performance was evaluated on out-of-sample classification accuracy, balanced between sensitivity (true positive rate), and specificity (true negative rate), and ROC AUC scores. Accuracy rate along with 95% confidence intervals for this rate were computed with a binomial test. P-values for classification performance were computed with a one-sided test, evaluating whether performance was better than the no information rate, taken to be the largest class percentage in the data. All above evaluation metrics were computed using the *caret* R package (Kuhn, 2008).

Classification coefficients of the individual DCT items, e, were computed by matrix multiplication

of the PCA rotation scores, w, and the model coefficients of the PCs, c:

$$\begin{bmatrix} e_1 \\ e_2 \\ \vdots \\ e_m \end{bmatrix} = \begin{bmatrix} w_{11} & w_{12} & \cdots & w_{1n} \\ w_{12} & w_{22} & \cdots & w_{2n} \\ \vdots & \vdots & \ddots & \vdots \\ w_{m1} & w_{m2} & \cdots & w_{mn} \end{bmatrix} \begin{bmatrix} c_1 & c_2 & \cdots & c_n \end{bmatrix} \tag{1}$$

where m is the individual DCT nouns (m=290) and n is the number of PCs included in the best model.

1.3 Post-hoc Data Sensitivity Analysis

To assess robustness of model performance and word effects against random variability in the training data, a post-hoc sensitivity analysis was performed with non-parametric bootstrapping. Each model was trained and evaluated on k=1000 new random partitions of the data into train and test sets (sampled with replacement). Model estimation and evaluation in each iteration followed the same procedure as described above for each model. Mean accuracy score across the 1000 data partitions, along with SDs for the accuracy rate, were computed for each model. Similarly, averaged classification coefficients of DCT items with associated SDs were computed across bootstraps.

1.4 Results: Classification Performance

The two DCT classification models performed significantly better than chance and outperformed the random baseline model. The mDCT model exhibited an accuracy of .61, 95% CI = [.56, .65], p¡.001 (accuracy = .61, 95% CI = [.58, .64], p¡.05 on training set). The mDCT+GenderAge model demonstrated a classification accuracy of .60 95% CI == [.55, .64], p¡.001 (accuracy = .66, 95% CI = [.63, .69], p¡.001 on training set). In comparison, the mGenderAge exhibited an accuracy of .57, 95% CI = [.53, .62], p¡.001 (accuracy = .60, 95% CI = [.57, .63], p¡.001 on training set), while the mRandomBaseline did not perform better than chance with an accuracy of .52, 95% CI = [.48, .57], p=0.17 (accuracy = .60, 95% CI = [.57, .63], p¡.001 on training set).

Bootstrapped robustness tests showed that both of the two DCT-models robustly performed better than the baseline model, while the mDCT+GenderAge robustly performed better than the mDCT model, with performance robustly above 60% accuracy (Figure 1. The mDCT model did not perform better than the mGenderAge model, in contrast to what was observed in the English study (Kruse, Rocca, & Wallentin, 2024), indicating that *gender* and *age* were more strongly associated with depression in these new six language samples than in the English sample. Results indicate however, that performance of DCT-based classification models in a sample including German, Spanish, Italian, Russian, Chinese and Filipino replicated that observed in the English study. This indicates that, across languages, the DCT elicits behaviors that allow inference on depression.

1.5 Results: Word effects

The fifty strongest predictive DCT items for each model are visualized in Figure 2 and 3. A positive regression coefficient indicates that participants in the depression group were more likely than individuals in the control group to select a proximal demonstrative for the given item, while a negative regression coefficient indicates that they were more likely to respond with a distal demonstrative compared to the control group. Bootstrapped robustness tests showed that the DCT items *shit*, *poverty*, *darkness*, *hell*, *emptiness*, *victim*, and *jealousy* were robustly the strongest positive predictors in both the mDCT and mDCT+GenderAge models. Contrary, the DCT items *sense*, *sport*, *office*, *team*, *sport*, *era* and *company* were robustly among the strongest negative predictors of depression in both models. These findings replicate the patterns observed in the English study, and recovers semantic effects of valence previously associated with depression (Liu et al., 2022; Eichstaedt et al., 2018; Chen et al., 2018; Preoţiuc-Pietro et al., 2015; Choudhury et al., 2013).

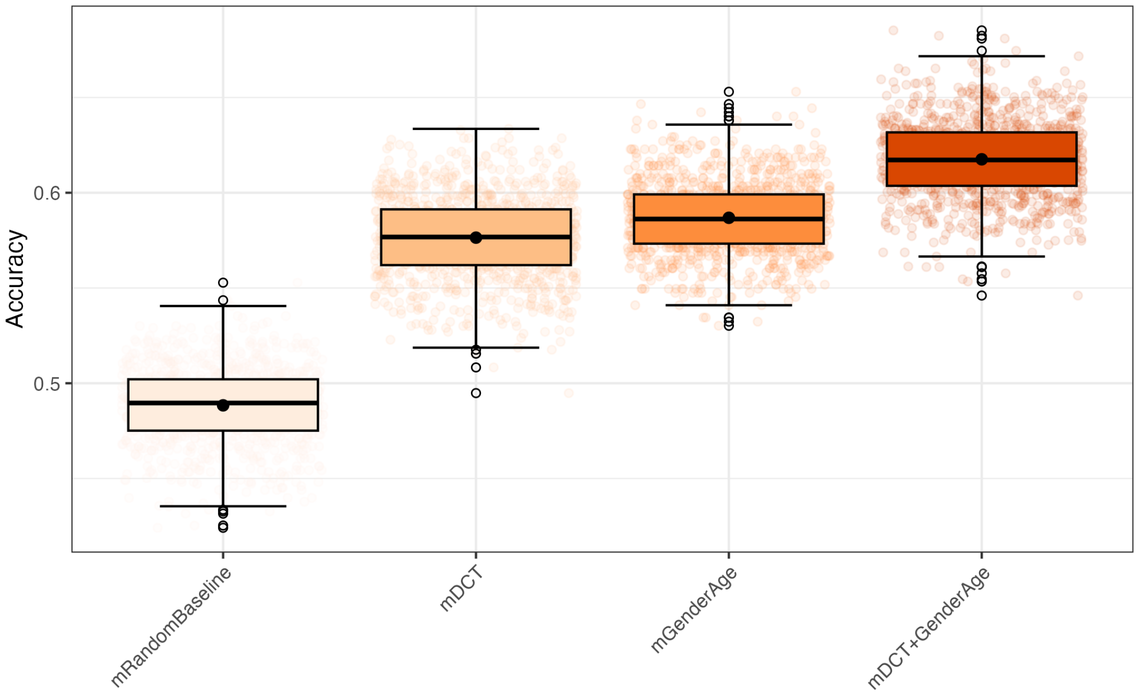

Figure 1: Bootstrapped performance (1000 bootstraps) of each model. Chance level is at 0.5.

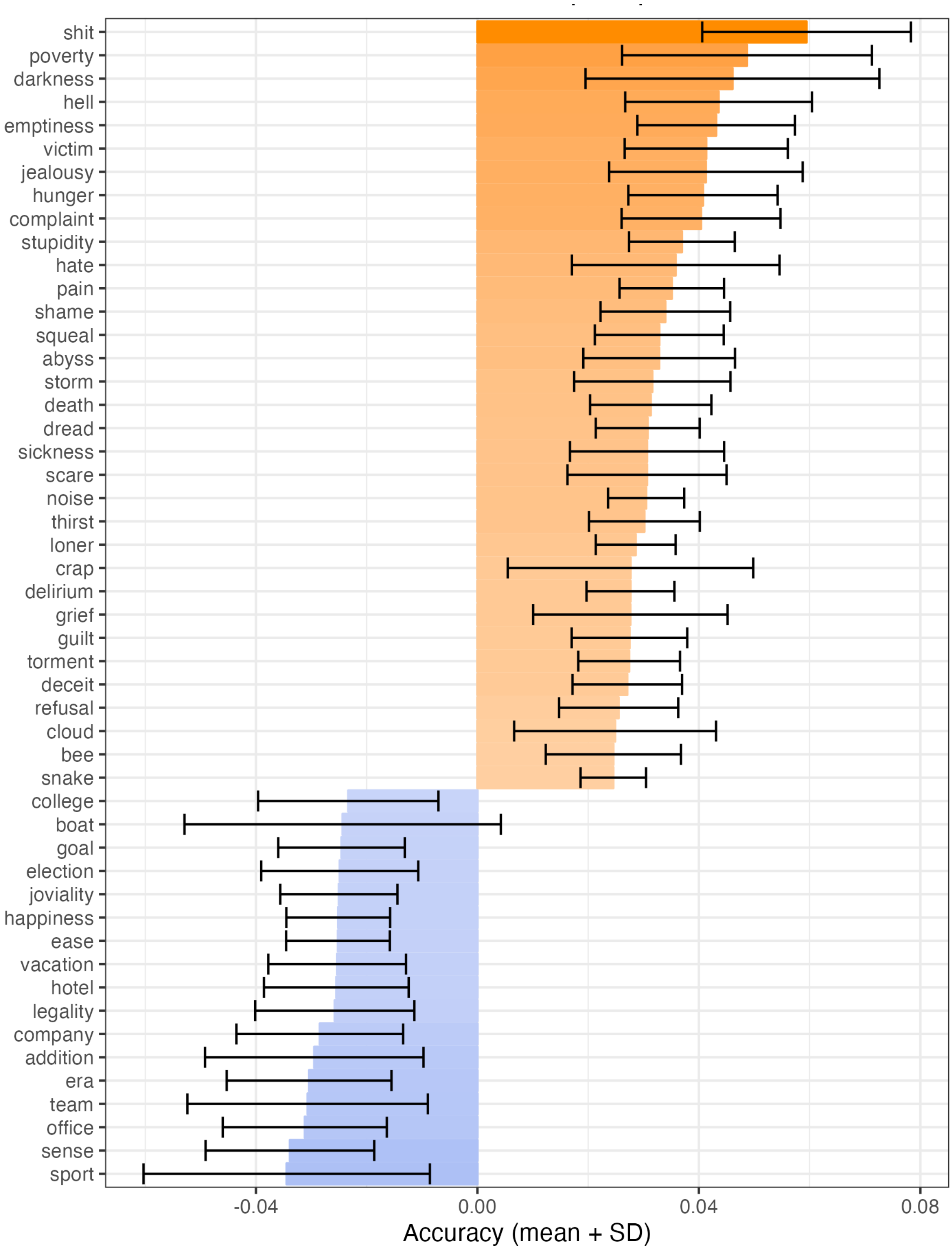

Figure 2: Bootstrapped classification coefficients of the top 50 predictive items in the mDCT model. Bars indicate mean coefficients across bootstraps and errorbars indicate the SD of coefficient estimates. Positive values indciate that the depression group were more likely to respond with proximal demonstratives for the given item compared to the control group, while negative values indicate that the depression group was more likely to respond with distal demonstratives for the given item, than the control group.

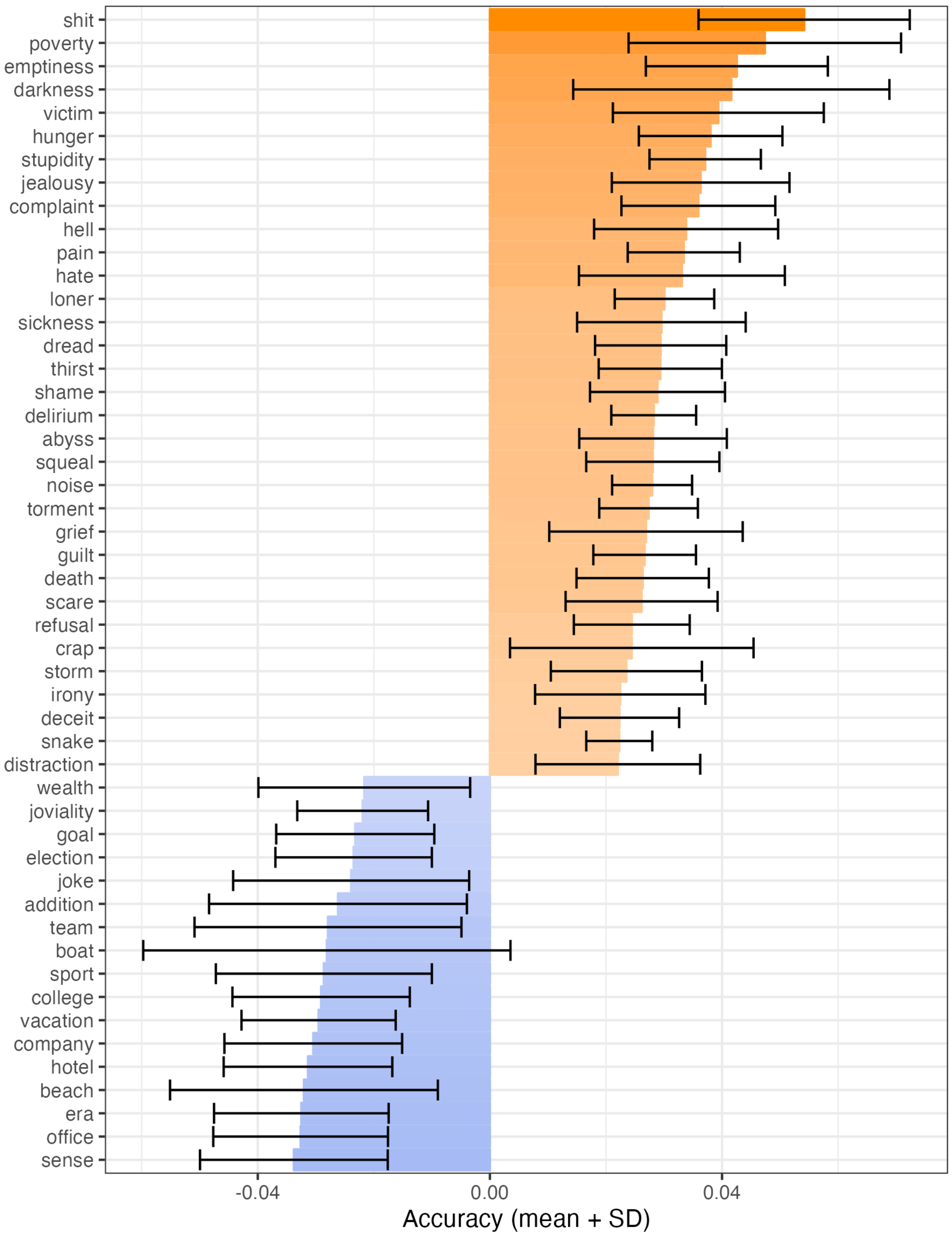

Figure 3: Bootstrapped classification coefficients of the top 50 predictive items in the mDCT+GenderAge model. Bars indicate mean coefficients across bootstraps and errorbars indicate the SD of coefficient estimates. Positive values indciate that the depression group were more likely to respond with proximal demonstratives for the given item compared to the control group, while negative values indicate that the depression group was more likely to respond with distal demonstratives for the given item, than the control group.

References

Chen, Xuetong et al. (Apr. 2018). “What about Mood Swings: Identifying Depression on Twitter with Temporal Measures of Emotions”. In: *Companion Proceedings of the The Web Conference 2018*. WWW ’18. Republic and Canton of Geneva, CHE: International World Wide Web Conferences Steering Committee, pp. 1653–1660. ISBN: 978-1-4503-5640-4. DOI: 10.1145/3184558.3191624. URL: https://doi.org/10.1145/3184558.3191624 (visited on 12/05/2022).

Choudhury, Munmun De et al. (2013). “Predicting Depression via Social Media”. en. In: *Proceedings of the International AAAI Conference on Web and Social Media* 7.1. Number: 1, pp. 128–137. ISSN: 2334-0770. DOI: 10.1609/icwsm.v7i1.14432. URL: https://ojs.aaai.org/index.php/ICWSM/article/view/14432 (visited on 09/01/2023).

Eichstaedt, Johannes C. et al. (Oct. 2018). “Facebook language predicts depression in medical records”. In: *Proceedings of the National Academy of Sciences* 115.44. Publisher: Proceedings of the National Academy of Sciences, pp. 11203–11208. DOI: 10.1073/pnas.1802331115. URL: https://www.pnas.org/doi/full/10.1073/pnas.1802331115 (visited on 10/28/2022).

Kruse, Line, Roberta Rocca, and Mikkel Wallentin (Mar. 2024). “Inferring Depression and Its Semantic Underpinnings from Simple Lexical Choices”. en. In: *Depression and Anxiety* 2024. Publisher: Hindawi, e3010831. ISSN: 1091-4269. DOI: 10.1155/2024/3010831. URL: https://www.hindawi.com/journals/da/2024/3010831/ (visited on 05/05/2024).

Kuhn, Max (Nov. 2008). “Building Predictive Models in R Using the caret Package”. en. In: *Journal of Statistical Software* 28, pp. 1–26. ISSN: 1548-7660. DOI: 10.18637/jss.v028.i05. URL: https://doi.org/10.18637/jss.v028.i05 (visited on 05/13/2022).

Kupfer, David J, Ellen Frank, and Mary L Phillips (Mar. 2012). “Major depressive disorder: new clinical, neurobiological, and treatment perspectives”. en. In: *The Lancet* 379.9820, pp. 1045–1055. ISSN: 0140-6736. DOI: 10.1016/S0140-6736(11)60602-8. URL: https://www.sciencedirect.com/science/article/pii/S0140673611606028 (visited on 12/05/2022).

Liu, Tony et al. (Apr. 2022). “The relationship between text message sentiment and self-reported depression”. en. In: *Journal of Affective Disorders* 302, pp. 7–14. ISSN: 0165-0327. DOI: 10.1016/j.jad.2021.12.048. URL: https://www.sciencedirect.com/science/article/pii/S0165032721013598 (visited on 07/19/2023).

Preoţiuc-Pietro, Daniel et al. (June 2015). “The role of personality, age, and gender in tweeting about mental illness”. In: *Proceedings of the 2nd Workshop on Computational Linguistics and Clinical Psychology: From Linguistic Signal to Clinical Reality*. Denver, Colorado: Association for Computational Linguistics, pp. 21–30. DOI: 10.3115/v1/W15-1203. URL: https://aclanthology.org/W15-1203 (visited on 08/31/2023).

Sap, Maarten et al. (Oct. 2014). “Developing Age and Gender Predictive Lexica over Social Media”. In: *Proceedings of the 2014 Conference on Empirical Methods in Natural Language Processing (EMNLP)*. Doha, Qatar: Association for Computational Linguistics, pp. 1146–1151. DOI: 10.3115/v1/D14-1121. URL: https://aclanthology.org/D14-1121 (visited on 12/05/2022).

Schwartz, H. Andrew et al. (Sept. 2013). “Personality, Gender, and Age in the Language of Social Media: The Open-Vocabulary Approach”. en. In: *PLoS ONE* 8.9. Ed. by Tobias Preis, e73791. ISSN: 1932-6203. DOI: 10.1371/journal.pone.0073791. URL: https://dx.plos.org/10.1371/journal.pone.0073791 (visited on 05/13/2022).

Team, RStudio (2020). *RStudio: Integrated Development for R*. RStudio, PBC, Boston, MA. URL: http://www.rstudio.com/.
