# Supplementary material for: This and that in depression: Cross-linguistic semantic effects
Source: PLOS Ment Health. 2025 Sep 24;2(9):e0000438. doi: 10.1371/journal.pmen.0000438 (PMC12798180; doi:10.1371/journal.pmen.0000438)
Supplement: S3 Supplementary Experimental Procedures — (PDF) [file pmen.0000438.s012.pdf]

S3 Supplementary Experimental Procedures

1 Extrapolation of Binder Scores

Following the procedure described in Turton, Vinson, and Smith, 2020, we used the 300-dimensional Glove semantic space (Pennington, Socher, & Manning, 2014) to extrapolate Binder scores for all 290 DCT items from their Glove vectors. A mapping between the 300-dimensional Glove space and the 65-dimensional Binder space was learned, and used to predict Binder feature scores on the 290 DCT items.

Six different models were trained to predict each of the 65 Binder scores from the respective Glove vectors on all the items in the Binder word set (535 words). These included a linear regression, Ridge regression, Random Forest, k-Nearest Neighbors, 2-layer neural network, and a 3-layer neural network. Models were trained for each of the 65 Binder features individually, and evaluated and compared on the average R2 and RMSE scores across all 65 features. On both metrics, the 3-layer neural network performed best across features (Figures 1 and Figure 2, see R2 scores for the best model on each of the 65 features in Figure 3).

To address the quality of predictions, the model was validated on the Lancaster Sensorimotor Norms (LSN) word set (Lynott et al., 2020) , including 31.455 words for which Glove vectors were available. As all Binder words are present in the LSN word set, and several of the features share high semantic similarities with Binder features (e.g., Binder-Audition/LSN-Auditory, Binder-Vision/LSN-Visual), the LSN word set provides a basis for evaluation of whether the model recovers the expected semantic structures between features (Turton, Vinson, & Smith, 2020). Note that all LSN words that were part of the Binder word set (and hence the training set) were excluded in computation of the following validation metrics.

First, the correlation vector between Binder features (one vector for each feature) were computed for the known word set (Binder) and the predicted word set (LSN). Then, the cosine similarity between correlation vectors of the same feature from the known and predicted scores was computed (e.g., vision(*predicted*) - vision(*known*), yielding a mean cosine similarity between vectors of the same feature of 0.97 (SD=0.02). In comparison, mean cosine similarity between correlation vectors of different features (e.g., vision(*predicted*)-pleasant(*known*)) were 0.07 (SD=0.10). This indicates that the predicted scores recovers the semantic relationship between Binder features.

Additionally, to assess whether the predicted Binder scores recovered the semantic relationships between Binder and LSN features, a correlation heatmap was computed between LSN and Binder features for a) the Binder word set (known Binder scores) and b) the LSN word set (predicted Binder scores). Results indicated that the predicted Binder scores for the $>$ 30.000 LSN words recovered the correlational structure between the LSN and Binder space, as was found in Turton, Vinson, and Smith, 2020 (Figure 4). The trained model was used to predict Binder scores for the remaining DCT items from the Glove vectors. The 30 DCT items scoring highest on each of the 65 semantic features are presented in Figures S5-S15.

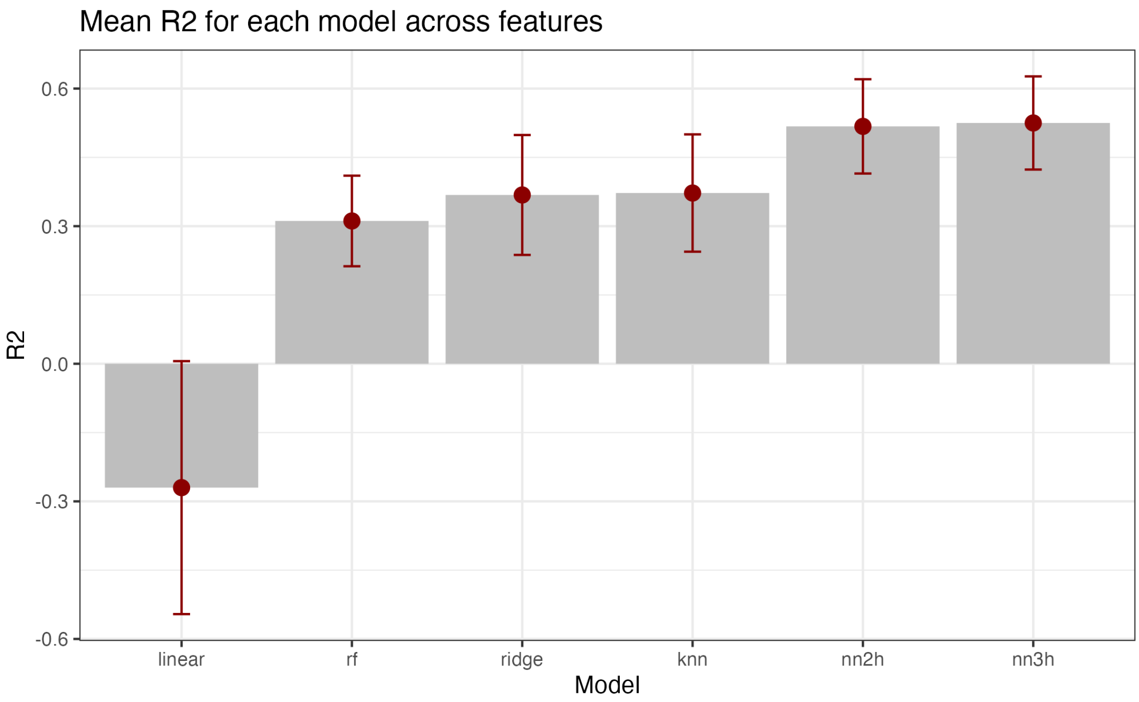

Figure 1: Performance of models (R2) predicting Binder scores from Glove vectors for all words in the Binder data set. Blue bars indicate mean R2 across the 65 models (one predicting each of the Binder features), and red bars indicate the associated SD.

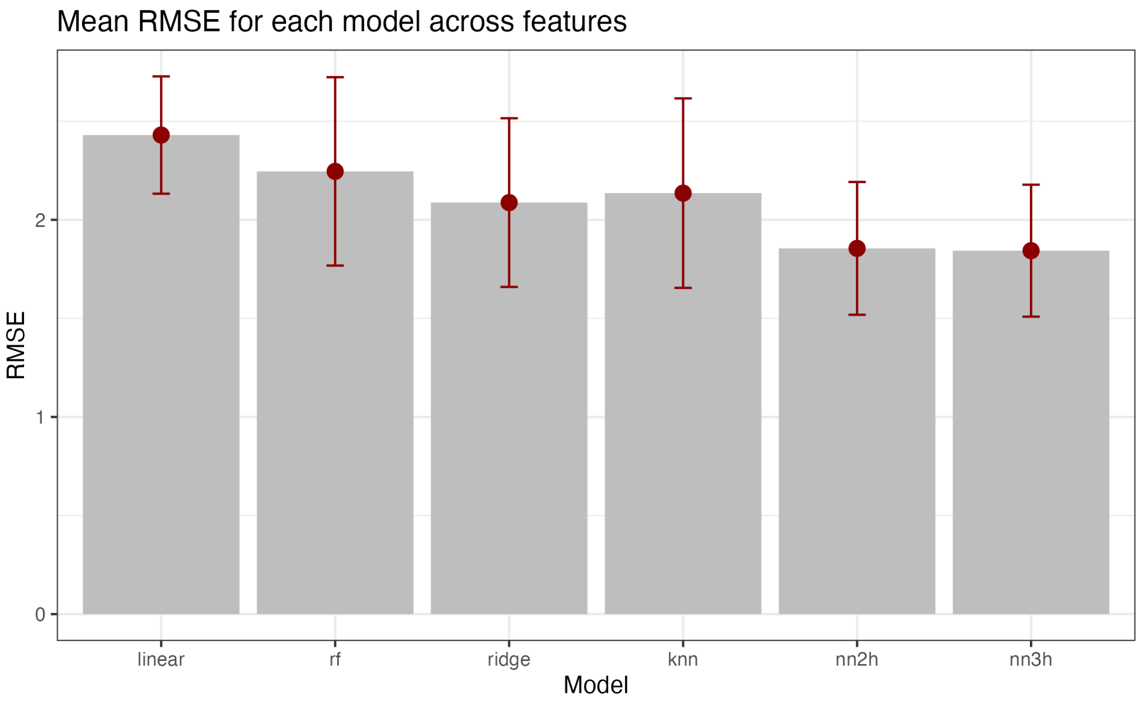

Figure 2: Performance of models (RMSE) predicting Binder scores from Glove vectors for all words in the Binder data set. Blue bars indicate mean RMSE across the 65 models (one predicting each of the Binder features), and red bars indicate the associated SD.

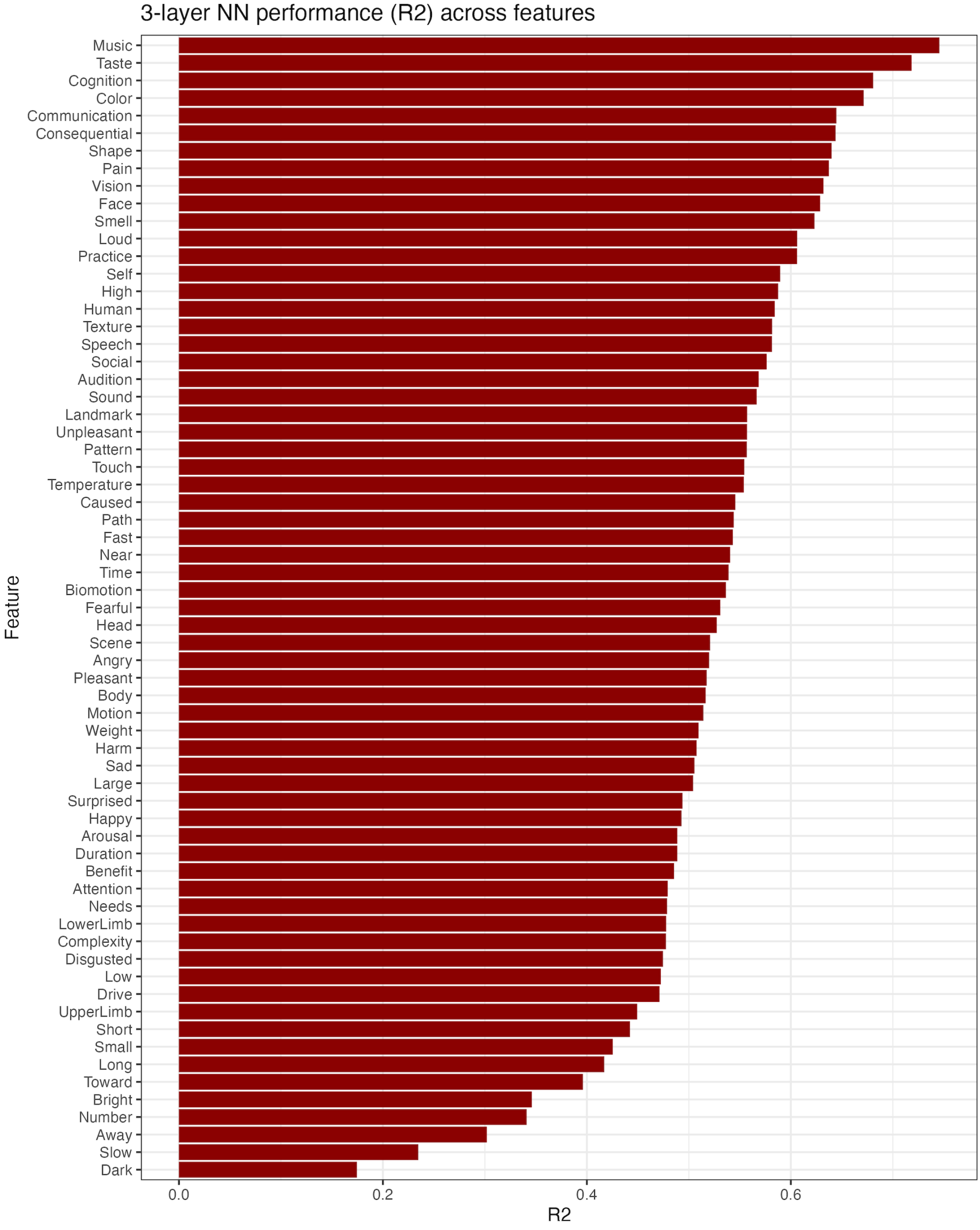

Figure 3: Performance (R2) of the best model (3-layer neural network) on each of the 65 Binder features.

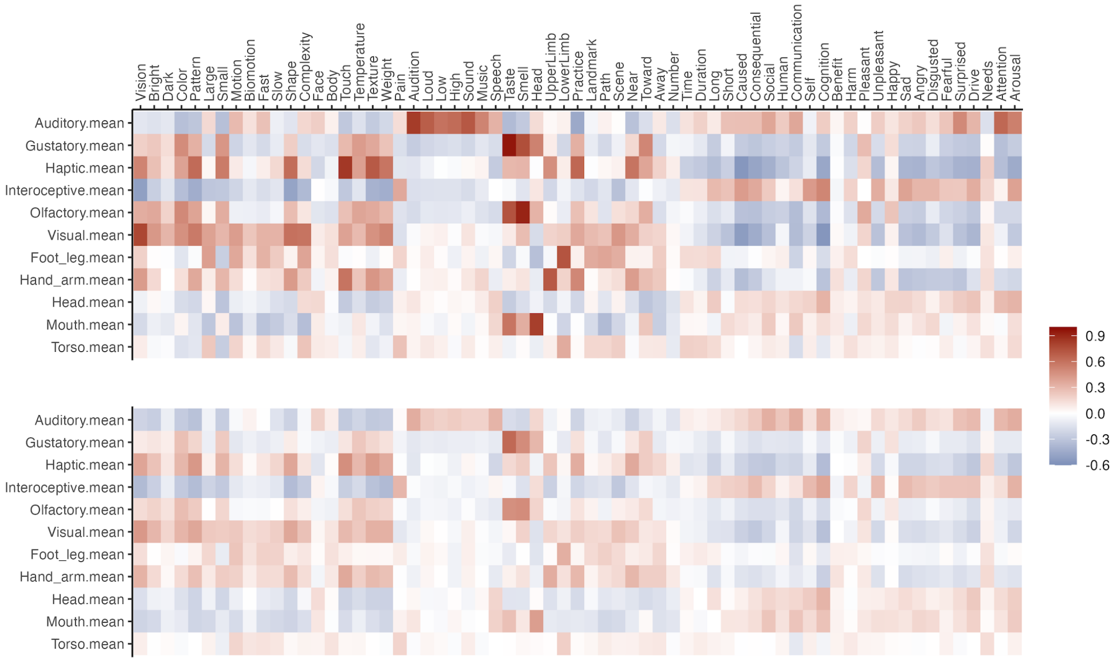

Figure 4: Correlation heatmap between LSN and Binder features for the Binder word set (known, top), and the LSN word set (predicted, bottom). Red values indicate higher correlation while blue values indicate lower values.

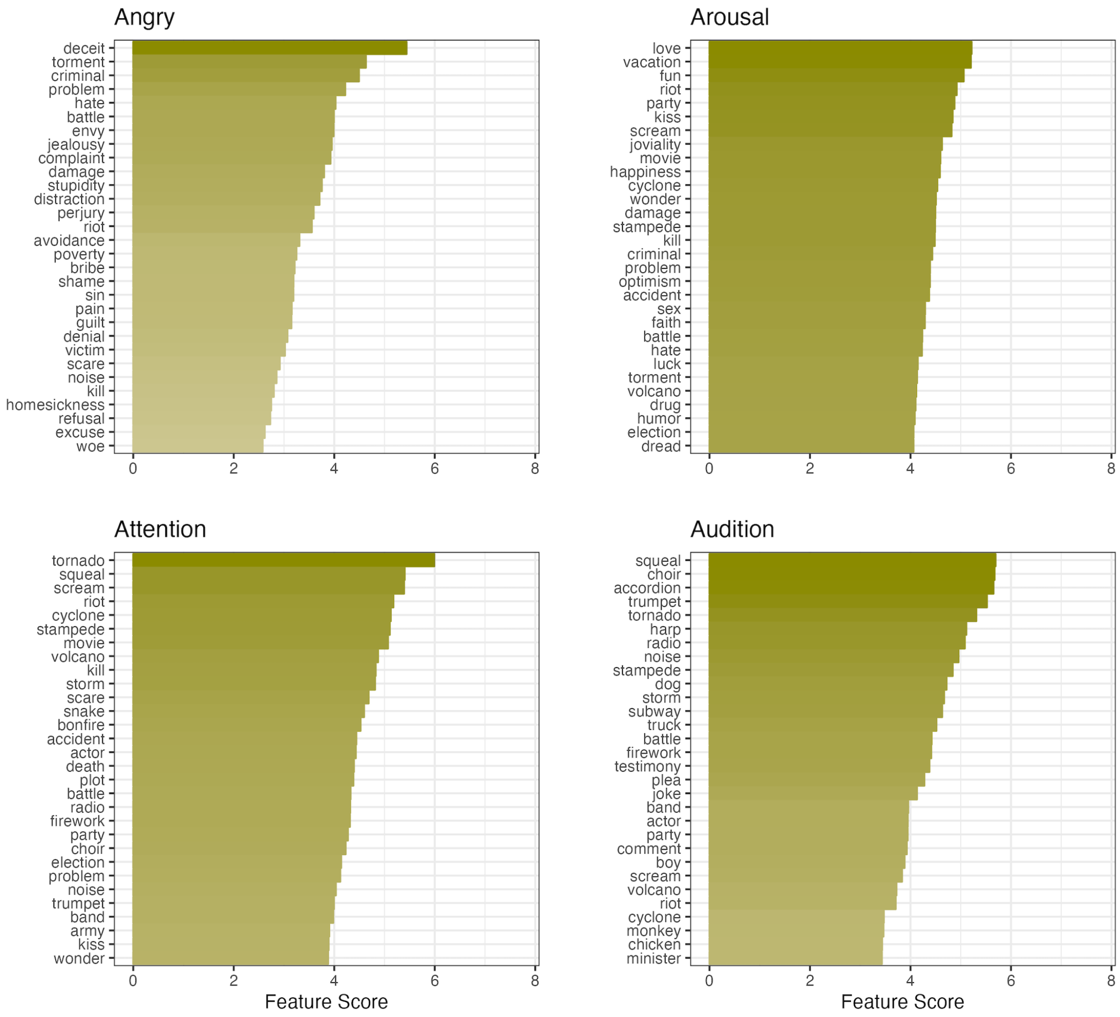

Figure 5: Features 1-6

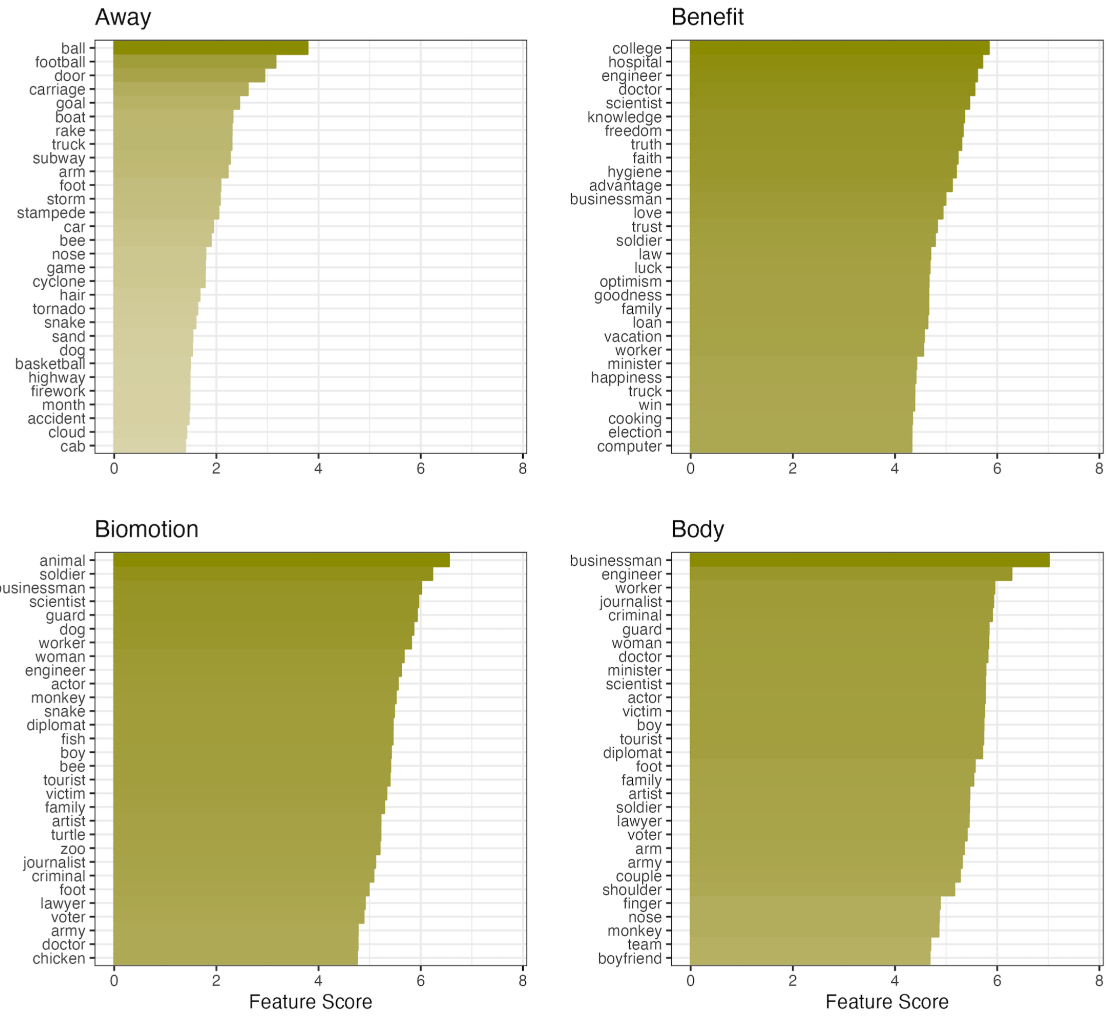

Figure 6: Features 7-12

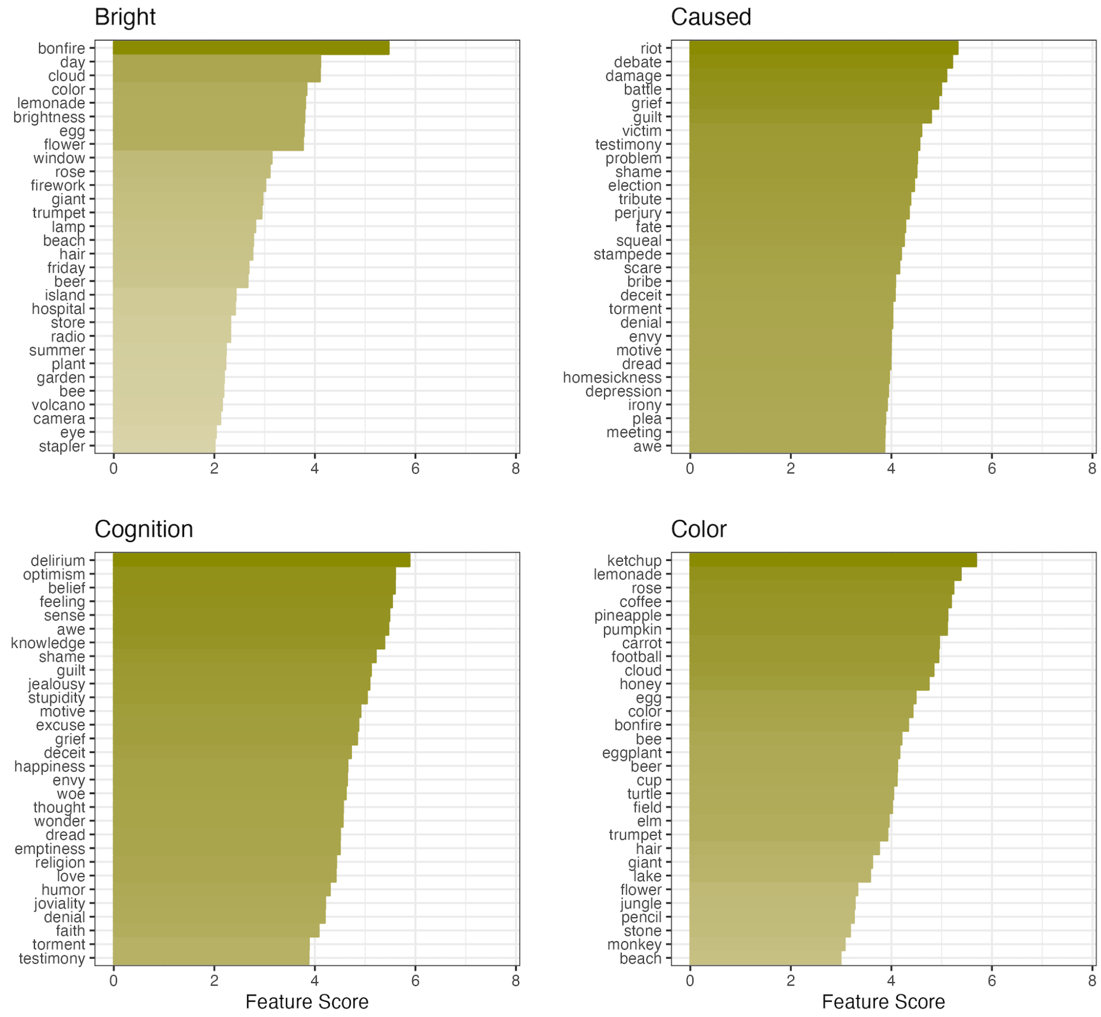

Figure 7: Features 13-18

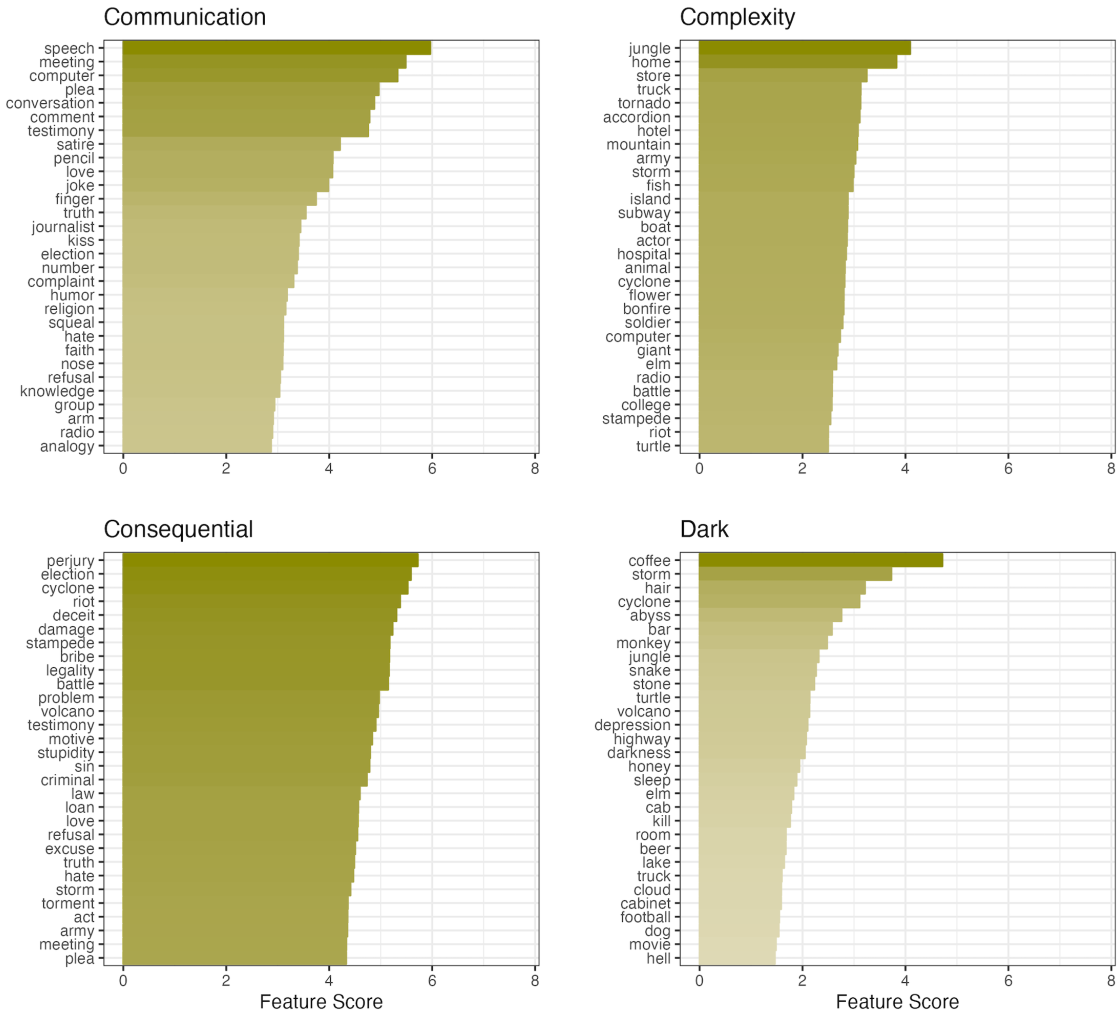

Figure 8: Features 19-24

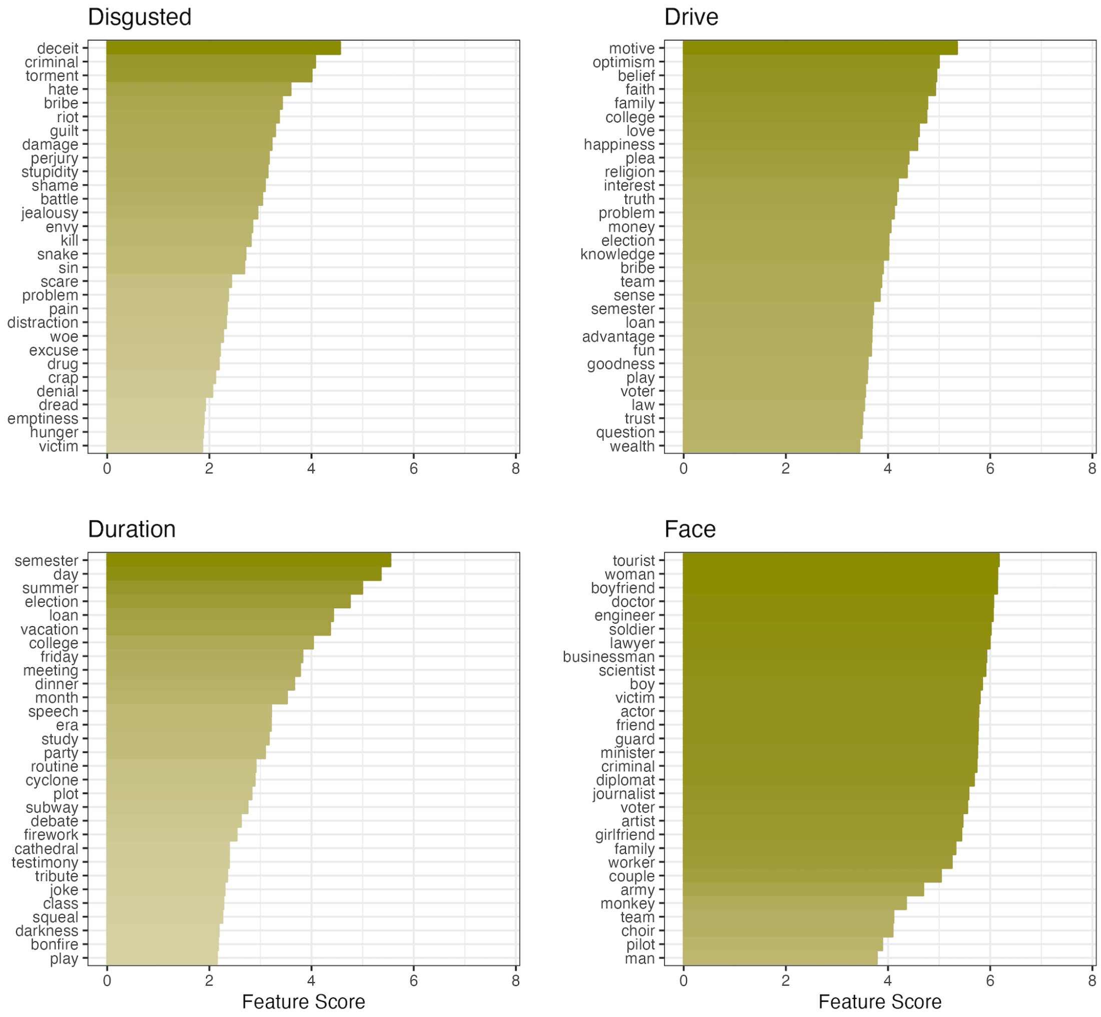

Figure 9: Features 25-30

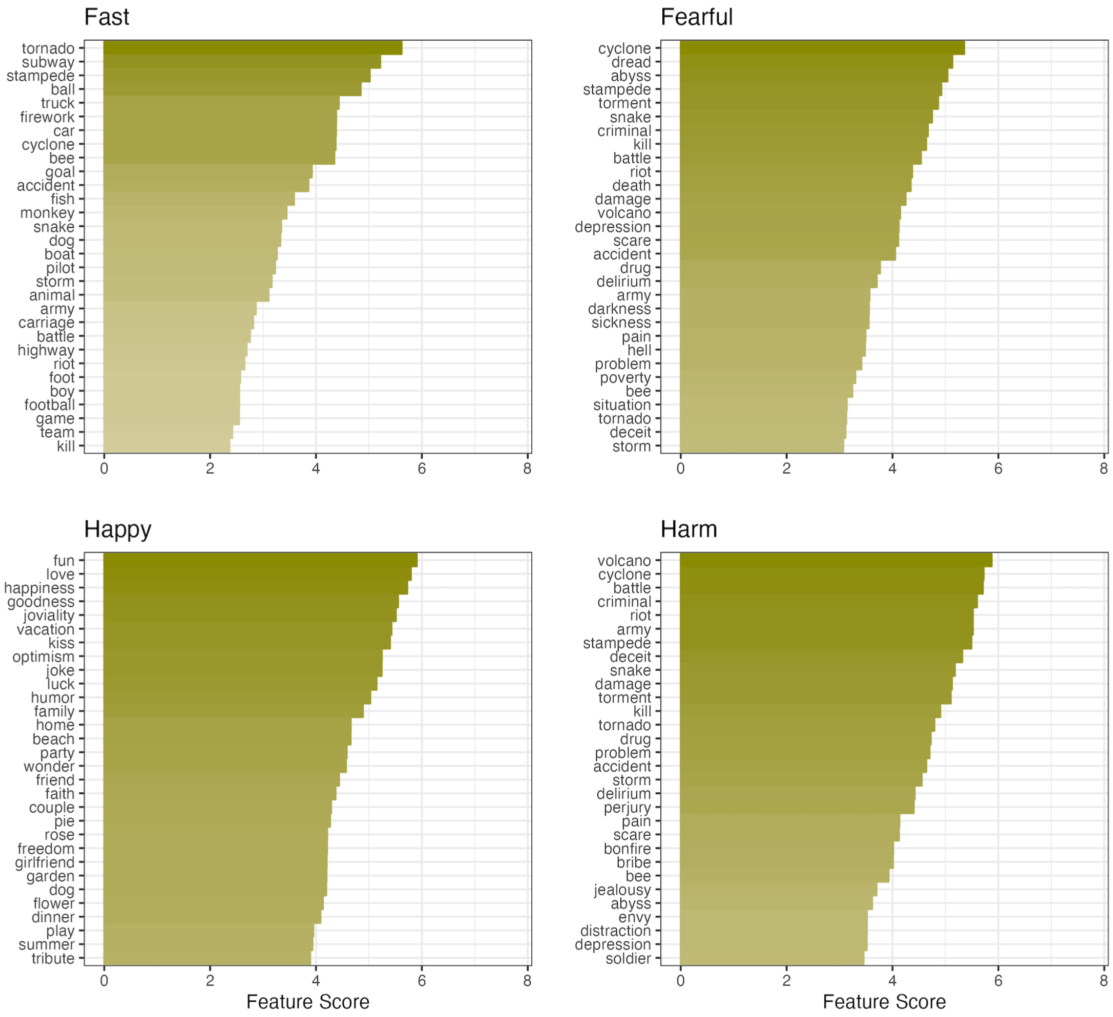

Figure 10: Features 31-36

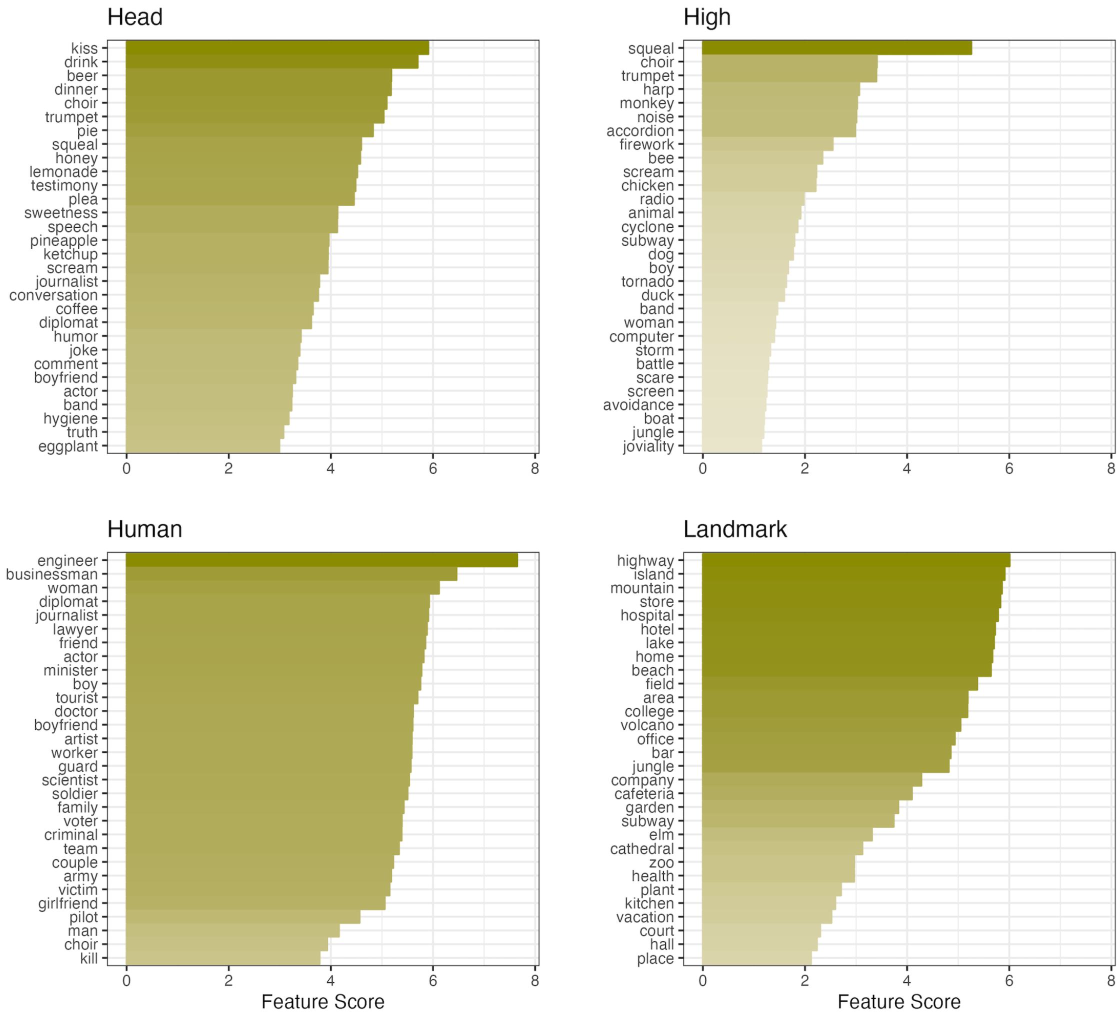

Figure 11: Features 37-42

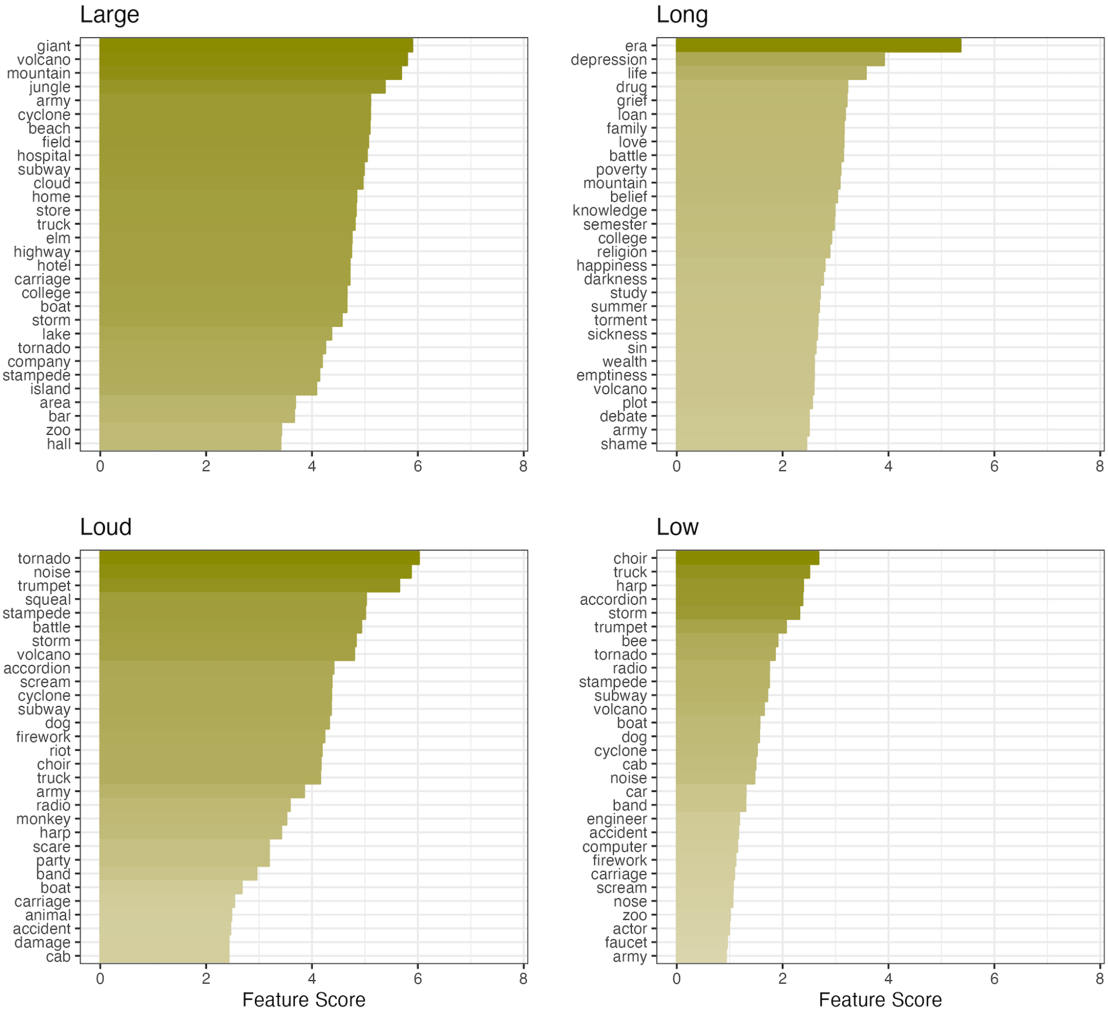

Figure 12: Features 43-48

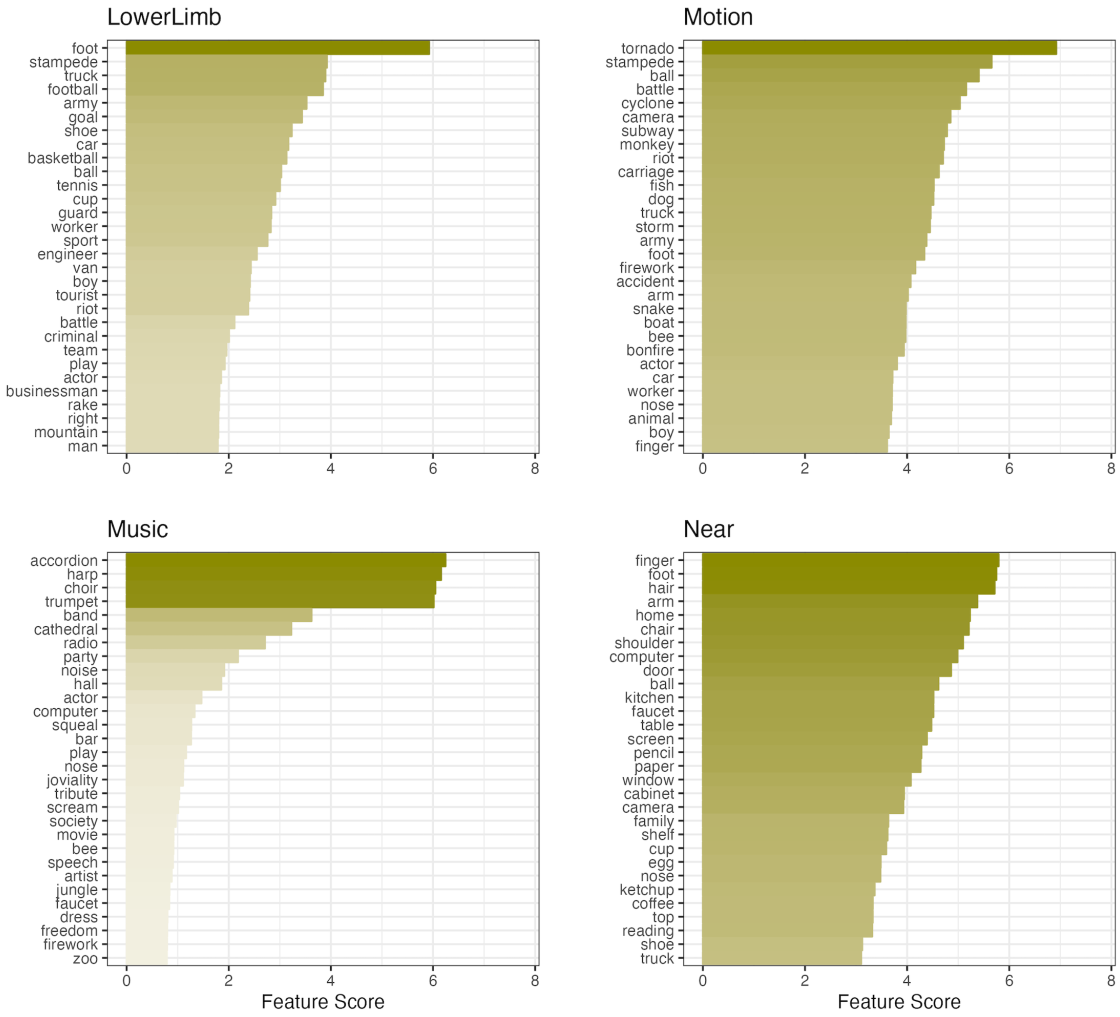

Figure 13: Features 49-54

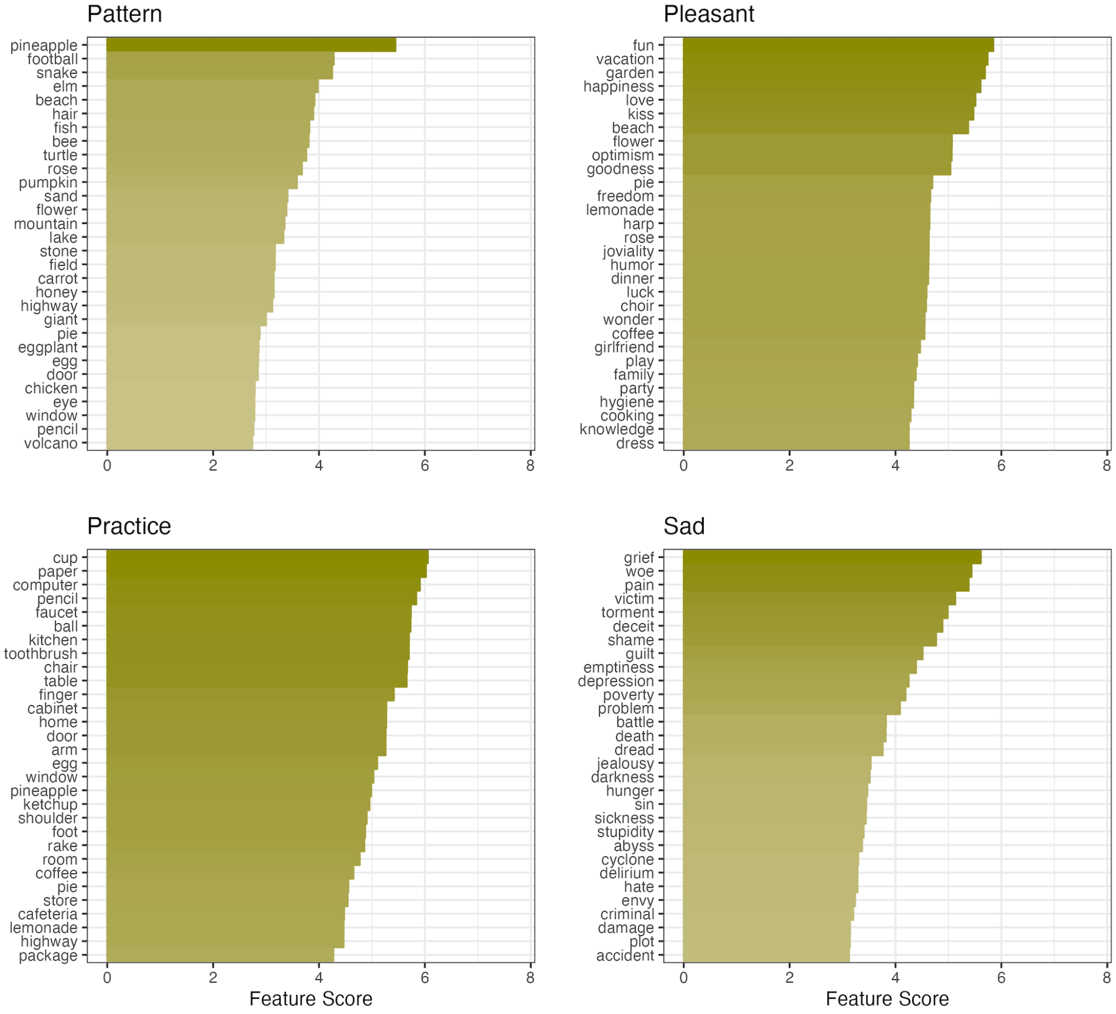

Figure 14: Features 55-60

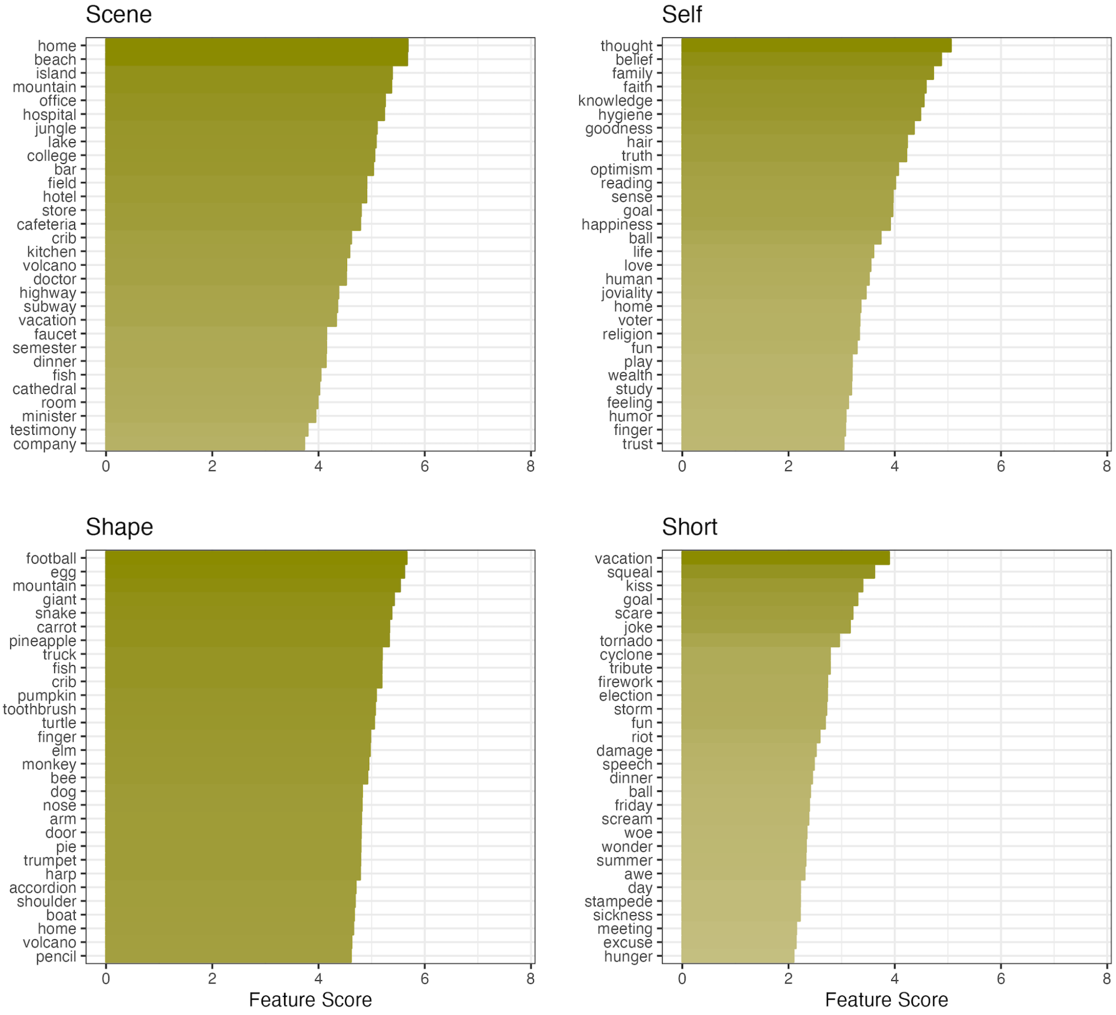

Figure 15: Features 61-65

References

Lynott, Dermot et al. (June 2020). “The Lancaster Sensorimotor Norms: multidimensional measures of perceptual and action strength for 40,000 English words”. en. In: *Behavior Research Methods* 52.3, pp. 1271–1291. ISSN: 1554-3528. DOI: `10.3758/s13428-019-01316-z`. URL: `https://doi.org/10.3758/s13428-019-01316-z` (visited on 06/11/2024).

Pennington, Jeffrey, Richard Socher, and Christopher Manning (Oct. 2014). “GloVe: Global Vectors for Word Representation”. In: *Proceedings of the 2014 Conference on Empirical Methods in Natural Language Processing (EMNLP)*. Ed. by Alessandro Moschitti, Bo Pang, and Walter Daelemans. Doha, Qatar: Association for Computational Linguistics, pp. 1532–1543. DOI: `10.3115/v1/D14-1162`. URL: `https://aclanthology.org/D14-1162` (visited on 06/11/2024).

Turton, Jacob, David Vinson, and Robert Smith (May 2020). “Extrapolating Binder Style Word Embeddings to New Words”. English. In: *Proceedings of the Second Workshop on Linguistic and Neurocognitive Resources*. Ed. by Emmanuele Chersoni, Barry Devereux, and Chu-Ren Huang. Marseille, France: European Language Resources Association, pp. 1–8. ISBN: 979-10-95546-52-8. URL: `https://aclanthology.org/2020.lincr-1.1` (visited on 05/06/2024).
