# Supplementary material for: This and that in depression: Cross-linguistic semantic effects
Source: PLOS Ment Health. 2025 Sep 24;2(9):e0000438. doi: 10.1371/journal.pmen.0000438 (PMC12798180; doi:10.1371/journal.pmen.0000438)
Supplement: S4 Supplementary Experimental Procedures — (PDF) [file pmen.0000438.s013.pdf]

S4 Supplementary Experimental Procedures

1 Comparison of models with different coding schemes for the medial demonstrative (in Spanish and Filipino)

To investigate the effect of the medial demonstrative form for classification performance, we compared the analysis excluding the medial demonstrative (yielding a binary proximal/distal distinction) to two additional models. The first contrasted proximal+medial vs. distal responses (proximal and medial = 1, distal = -1), and the second contrasted proximal vs. medial+distal responses (proximal = 1, medial and distal = -1). Semantic profiles for each participant were recomputed using each of these response coding schemes, and Decision Tree Ensemble classification models were trained and evaluated as described in the methods section of the paper.

1.1 Spanish results

Results of the Spanish model comparison indicated that the model excluding the medial responses (-*m*) and the model contrasting proximal vs. medial+distal (*md*) both outperformed the baseline model. The -*m*-model performed best (mean F1=0.59, SD=0.05), followed by the *md*-model (mean F1=0.58, SD=0.05). The model contrasting proximal+medial vs. distal performed worst (mean F1=0.55, SD=0.05) and showed bootstrapped F1 distribution overlapping with the baseline model (Figure 1). Pairwise rank correlations of feature effects between the -*m*-model and the *md*-model was 0.64, and between the -*m*-model and *pm*-model it was 0.42 (Figure 2). For the *md*-model and the *pm*-model feature effects showed a rank correlation of 0.23. Thus, both model performance and importance ranks of semantic features do not seem to be influenced much by excluding the medial responses compared to coding them as distal, while coding them as proximal causes a reduction in performance. These results suggests that the behavioral differences associated with depression are expressed more similarly in the choice of medial and distal demonstratives versus proximal demonstratives. Further, the fact that model performance is best when excluding the medial demonstrative, indicates that the group differences in medial response behavior may be smaller, compared to the group differences in proximal and distal response behavior.

1.2 Filipino results

Results of the Filipino model comparison showed that the -*m*-model and the *pm*-model both outperformed the baseline model. The *pm*-model performed best (mean F1=0.64, SD=0.08), followed by the -*m*-model (mean F1=0.62, SD=0.08). The *md*-model performed worst (mean F1=0.56, SD=0.09) and showed bootstrapped F1 distributions largely overlapping with the baseline model (Figure 1). Pairwise rank correlations of feature effects for the -*m*-model and the -*md*-model was 0.67 and for the -*m*-model and the *pm*-model it was 0.49 (Figure 3). Rank correlation coefficients of feature effects between the *md*-model and the *pm*-model was 0.35. These results indicate that in the Filipino case, behavioral differences related to depression are expressed more similarly in the proximal and medial responses. Further, excluding the medial responses causes a drop in performance and larger difference in feature effects compared to the best model, indicating larger group differences in medial response behavior in the Filipino data, compared to the Spanish data.

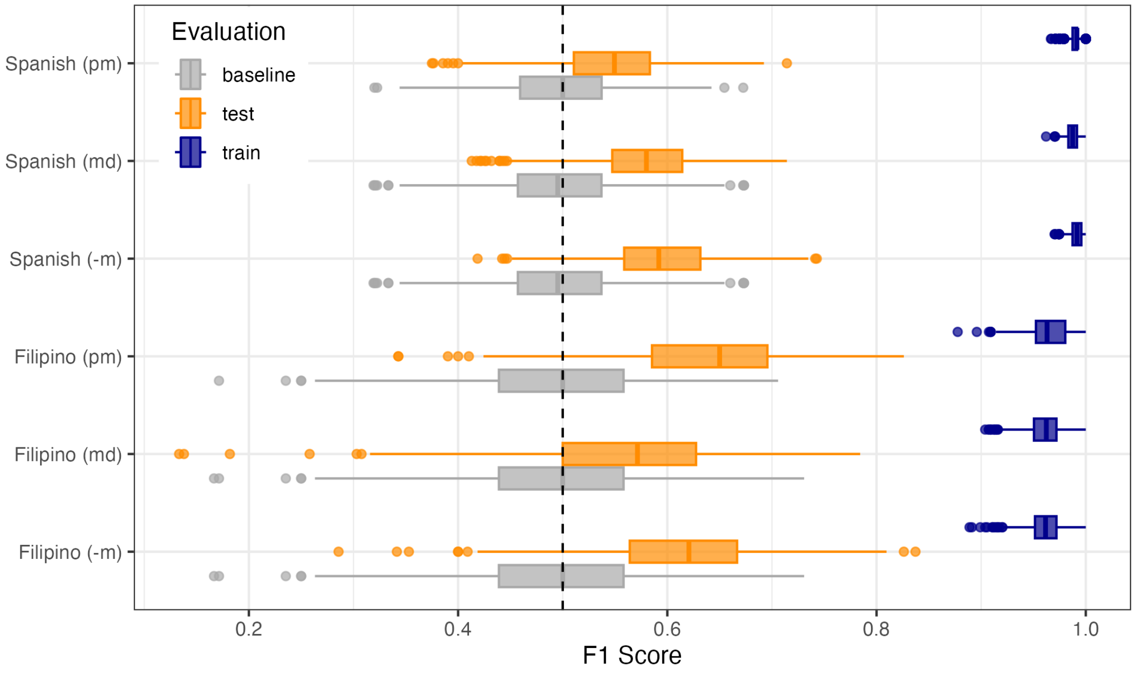

Figure 1: Bootstrapped performance (F1) distribution of the three compared Spanish and Filipino models.

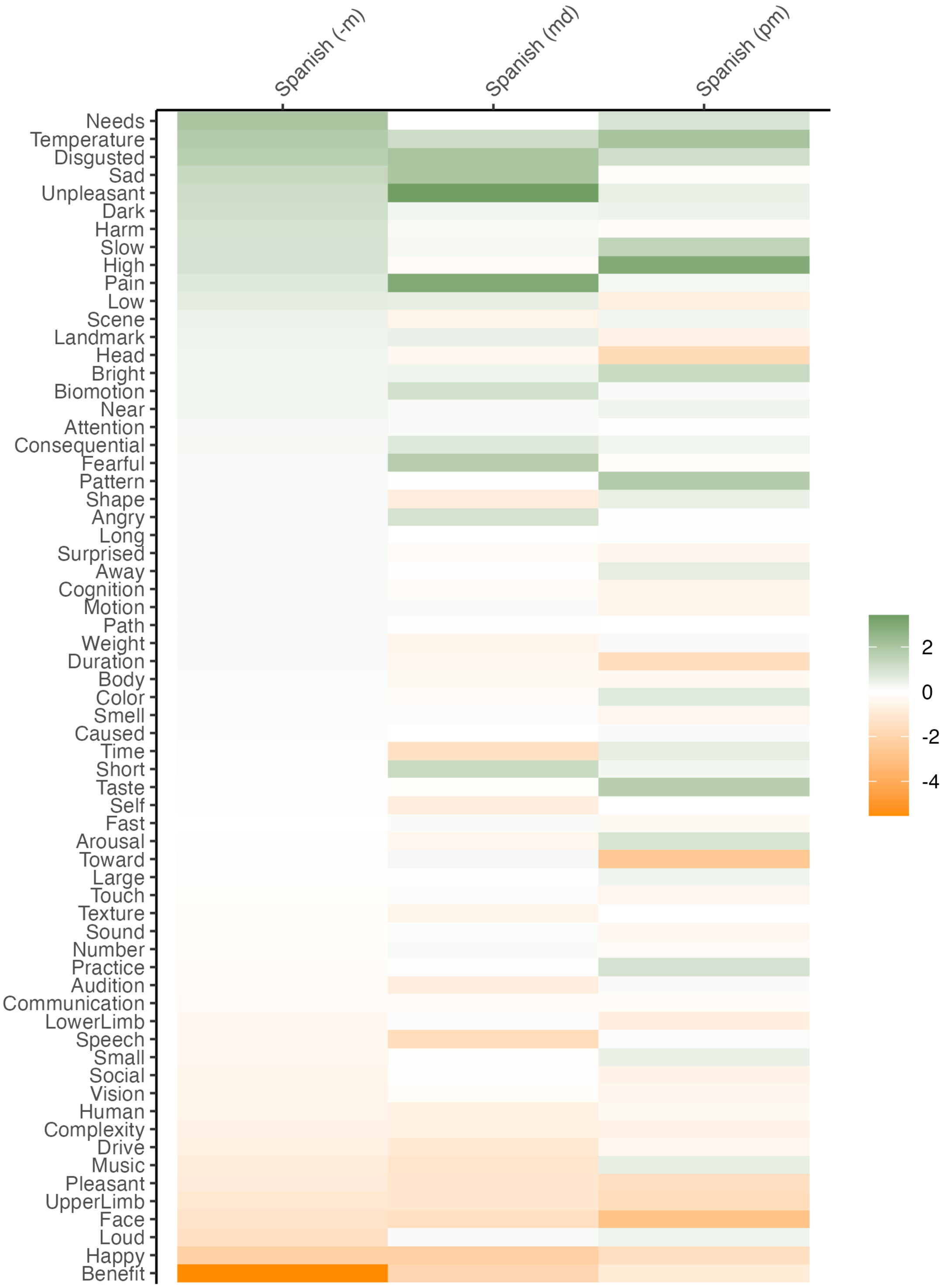

Figure 2: Comparison of feature effects (SHAP) between the three Spanish models of different medial coding schemes.

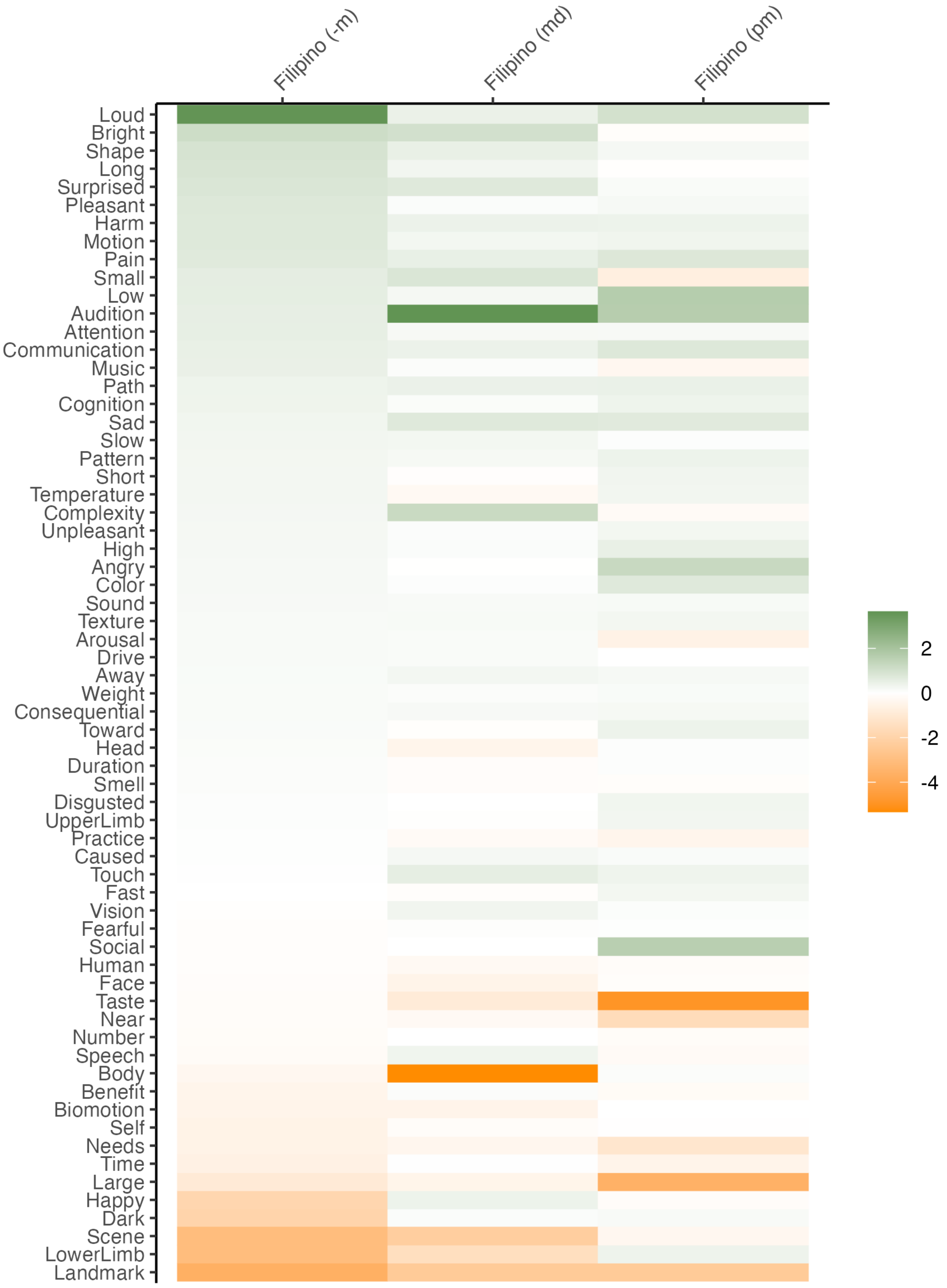

Figure 3: Comparison of feature effects (SHAP) between the three Filipino models of different medial coding schemes.
