# Supplementary material for: This and that in depression: Cross-linguistic semantic effects
Source: PLOS Ment Health. 2025 Sep 24;2(9):e0000438. doi: 10.1371/journal.pmen.0000438 (PMC12798180; doi:10.1371/journal.pmen.0000438)
Supplement: Attachment — Submitted filename: Response_to_reviewers_10-01-25.pdf [file pmen.0000438.s014.pdf]

Resubmission: Response to comments

Line Kruse
Ph.D. Student
Dept. of Linguistics, Cognitive Science and Semiotics, Aarhus University
Jens Chr. Skous Vej 2, 1485-637, 8000 Aarhus, Denmark
linekruse@cc.au.dk
+45 61650200

10.01.25

Dear Editorial Board,

This is a resubmission of the manuscript, previously submitted with the submission number PMEN-D-24-00372. The manuscript was automatically withdrawn due to missing updates that were requested by you before the revision deadline. The automatic withdrawal, however, happened before the deadline I was informed of, which is the reason that it was not revised in due time. The requested revision of the methods section to include date and time of data collection has been updated in the present submission.

Please address all correspondence concerning this manuscript to me at linekruse@cc.au.dk

Sincerely,

Line Kruse
