# Supplementary material for: This and that in depression: Cross-linguistic semantic effects
Source: PLOS Ment Health. 2025 Sep 24;2(9):e0000438. doi: 10.1371/journal.pmen.0000438 (PMC12798180; doi:10.1371/journal.pmen.0000438)
Supplement: Attachment — Submitted filename: Response_to_reviewers_04-07-25.pdf [file pmen.0000438.s015.pdf]

PMEN-D-24-00582

Response to Reviewers

02.07.25

Line Kruse, Ph.D
Dept. of Linguistics, Cognitive Science and Semiotics, Aarhus University
Jens Chr. Skous Vej 2, 1485-637, 8000 Aarhus, Denmark
linekruse@cc.au.dk
+45 61650200

Dear Editor and Reviewers,

We thank you very much for the careful and detailed comments provided in your review of the paper. We agree on the issues raised in the review and believe that the corresponding revisions have significantly increased the quality of the paper. Below, we outline responses to the comments and edits done to accommodate the issues raised.

1 | Technical and formatting issues

All noted typos and misspellings identified by both reviewer 1 and 2 have now been corrected. Further, reviewer 1 noted that some paragraphs were too long. These have now been identified throughout the paper and broken down into shorter sentences or rephrased.

Reviewer 2 noted that some of the references were missing the *year* in the bibliography. This was in fact the case for all references and was caused by an error in the library import from Zotero. The error was identified and corrected, and all references are now in correct format including the *year* field.

Following reviewer 1's comment that tables 1, 2 and 5 were formatted as figures, all tables in the manuscript have now been formatted as formal latex tables. To make the tables fit on the page, we performed some reformatting of the tables. For table 1, this included splitting it into two sections, that are now placed on top of each other. For table 2, reformatting included renaming the column "Response options" to "Options", "Proximal" to "Prox", "Distal" to "Dist" and "Medial" to "Med". We have instead added descriptions of these shortened terms in the table caption. Table 2 did not fit into the page with adjusted width set to -2.5 inches (cf. the PLOS submission guidelines). It is currently adjusted to -2.7 inches allowing it to fit on the page, however, if the table can be slightly reduced in size (or font size), this will provide a better solution.

To accommodate reviewer 2's suggestion of a figure illustrating the experimental paradigm, we have now removed Table 4 to allow space for an extra figure exemplifying two trials in the task. Table 4 is instead added as a supplementary item labelled S1 Table and referred to in the text. We believe that this is an improvement, as the new task illustration figure is more relevant and useful than Table 4, which could easily be degraded to supplementary information. Thank you very much for this suggestion.

It was commented by reviewer 2 that some of the supplementary figures were of rather low quality. To accommodate this issue, all supplementary figures have been remade and updated to the required resolution (at least 300 dpi) and size. For some figures this required using a different library for plotting, which means that they will have a slightly different visual format than in the previous submission. The content, however, remains identical.

2 | Clarification, explanation and discussion issues

Reviewer 1: Although the cross-linguistic validity of the proposed method is raised as a concern in the limitation section, lines 182-183 still justify that hte Binder "is not language specific". As the authors acknowledge, the results instead suggest a cross-linguistic limitation of this method. These lines should be removed or modified.

Reviewer 1 made the very useful comment that the statement "*the Binder ratings used in the present study is not language specific*" (line 182-183) was misleading, as it is later (in the discussion section) discussed exactly how the use of these English-based ratings is a limitation. While this line was intended to make clear that the ratings are based on the English language and are thus not specific to the other languages compared in the paper, we see how this formulation was ambiguous and could be misinterpreted as stating that the ratings can be considered "general" across languages. This is a very important point to get clear in the paper, and the line has been edited to remove any ambiguity. It now reads "*Note that the semantic Binder scores included in the present study are based on human ratings of English words*" (line 192-193).

Reviewer 2: For better understanding, could you explain what "contextual anchors" are? I can imagine, but would be great to have an example.

To clarify what is meant by “contextual anchors” as suggested by Reviewer 2, we have now added a short paragraph in the beginning of the section where the term is first mentioned. It now reads “*In normal communication, the above factors as relative proximity to the referent, relative position of the interlocutor, or shared familiarity with the referent, act as contextual anchors guiding the choice of demonstrative form depending on the communicative context or goal. For instance, a speaker may use the proximal demonstrative (“is* this *my cup?”) to distinguish it from another cup slightly further away and reduce ambiguity about which cup is being referred to”* (line 36-41). This should provide an explanation of what is meant by the term as well as an example of how it would “anchor” demonstrative choice in normal communication. As such, the contrast to how it is used in the DCT (experimental paradigm) should be clearer and more comprehensible.

Reviewer 2: Not 100% sure about the experimental setup of DCT - the participants get one word and then they have to associate it with eihter “this” or “that”, is that correct? Also here, it would be great to see an example or a screenshot of the interface the participants are dealing with

The revised paper now includes, in addition to the added figure illustrating two trials in the paradigm, an example written in text where the paradigm is outlined (line 46). This was indeed a very important point, and this change should reduce uncertainties about the experimental paradigm.

Reviewer 2: Could you give an example of how participants were assigned Binder features exactly?

Reviewer 2 suggested a clarification and example of how exactly the semantic (Binder) feature scores for each subject were calculated. To accommodate this, we have now added a paragraph providing an example in the section outlining the calculation process. It reads “*For instance, the three words “party”, “car”, and “shame” score 5.9, 2, and 2.5 on the semantic dimension* social, *respectively. On the semantic dimension* sad, *they score 0.03, 0.1, and 4.8, respectively. Say subject A responded with “this” (1), “that” (-1) and “that” (-1) to the three words, respectively. The mean score for subject A on the semantic feature* social *would then be ((1*5.9)+(-1*2)+(-1*2.5))/3 = 0.5. The mean score for subject A on the semantic feature* sad *would be ((1*0.03)+(-1*0.1)+(-1*4.8))/3 = -1.6. This was computed across all 290 words in the task for all 65 semantic features, yielding a 65-dimensional semantic vector for*

each subject based on their demonstrative choices" (line 233-244). This section follows immediately after the methodological explanation of this procedure and should hopefully clarify exactly how these scores can be interpreted.

Reviewer 2: Could you explain more clearly why you did not use the DCT responses directly for classification?

This is indeed a highly important aspect of the paper and we are grateful for the comment. Since classification based on the (word-level) DCT responses was conducted in previous work (Kruse et al., 2024), which is cited in the present paper, and this is what is also conducted in the first part of the current analysis (the replication analysis), it was unclear whether this comment meant 1) It is not clear that the replication analysis is using the raw DCT based responses, or 2) It is not clear why the classification models using DCT responses (in previous work and in current replication) is based on PC representations of the responses (PCA). We have thus implemented changes to accommodate both possibilities. First, the last paragraph of the "Present Study" section has been edited, to make it more clear that the replication analysis is based on DCT responses directly (line 114-116). Additionally, we have added information to the "Classification by Semantic Profiles" section, to clarify why subject-specific aggregated semantic scores were used in the present paper and how they are different (and relevant) compared to previous analyses. The edited section now reads: "*the original English study and the replication analysis in the 6 new languages used item-level responses as input for the depression classification. Results indicated specific DCT words that were associated with robust differences in demonstrative choice behaviour predictive of depression symptom severity. If these patterns indeed reflect general semantic differences, semantically similar words should elicit similar effects. Contrary, it may be that previously observed effects pertained to only to the specific words in the test, rather than general semantic differences. To investigate the extent to which depression related differences in demonstrative choice reflect differences along generalized semantic dimensions, beyond individual words, we used subject-specific semantic scores aggregated across choices for all items as input to the classification model. That is, for each subject, a semantic profile was computed, based on their responses across all items in the DCT. If the differences between the depression and control groups reflect a word-effect, rather than a semantic effect, aggregated semantic input features (across words) should yield little effect in the classification model and model performance should be poor. Contrary, if the differences in*

responses are consistent along specific semantic features, then these features should yield large predictive effects in the classification model. In the latter case, this would suggest that there are semantic dimensions for which demonstrative reference are predictive of depression, potentially indicating semantic characteristics of altered psychological space" (line 210-228). This section is followed by an updated "Input Features" section, describing (with example) how exactly the subject-specific semantic feature scores were calculated (see above).

To clarify why the replication analysis based directly on DCT responses employed PC representations of the DCT responses (computed with PCA), we have added a line in the section "Replication of English Analysis", explaining that this was done (and necessary) to reduce dimensionality of the input space and avoid issues of multicollinearity for statistical analyses (the logistic regression models would otherwise have had N words (= 300) predictors, some of which are highly correlated) (line 199-200). The methods section on this analysis also refers to the previous work, of which this analysis is a replication, where these analyses are described in greater detail.

Reviewer 2: Since the data is very imbalanced and also because the diagnosed group is more relevant in this task, it would be more interesting to see performance (e.g., F1) of the positive class, additionally to the scores across classes.

We agree that these metrics are interesting to have in addition to, and in comparison to, the cross-class metrics. Indeed, Table 3 and Table 4 include precision and recall metrics, which pertain specifically to model performance on the positive class. However, in the former version, these were not highlighted or discussed appropriately, as pointed out by the reviewer. To accommodate this, we have added the following paragraph to the methods section describing the model estimation procedure, to more directly point attention to those scores: "*F1 and ROC-AUC metrics on each test-set indicate classification performance across classes. Precision metrics indicate the fraction of cases classified as depression that were true cases of depression, while recall metrics indicate the fraction of positive cases (depression) that were indeed classified as depression by the model"* (line 286-290). These however, must be interpreted in relation to the cross-class F1 scores, as a recall of 1.0 could technically be obtained by the model always predicting the positive class. The edits in this section should now draw attention to performance metrics of the positive class only and allow

more direct comparison and interpretability of model performance. We thank Reviewer 2 very much for making us aware that this information was not clearly reported.

Reviewer 2: "suggests that the model learned patterns associated iwth depression that are largely shared across languages". It owuld be great to see an analysis of what these patterns are/might be

It was pointed out by Reviewer 2 that while the discussion states that results suggest that "the model learned patterns associated with depression that are largely shared across languages", it is not clear from that what these are and suggested an analysis of this. This statement was intended to refer to the analysis of the feature importances (SHAP values) and their correlation across models and languages, from which we can see the extent to which the most important semantic features for the classification are shared or different. Particularly, the feature importances of the cross-linguistic model (Figure 4) provides indications on patterns that are shared across languages (since results also indicated that this model performed well on out-of-sample predictions in each language, except for Chinese). Additionally, the comparison of feature-importances across models for each language (Figure 5 and 6) indicate similarities and differences in the semantic patterns associated with depression between models/languages. The details of these results and the patterns they capture that distinguish the two groups, are discussed together in the discussion section line 480-509. However, to clarify that the commented statement refers to these results, we have added a short paragraph anticipating this discussion in the section where the statement is written. It now reads: *"these results suggests that the model learned patterns associated with depression that are largely shared across languages. As discussed in detail further below, these seems to be more frequent use of the proximal demonstrative for semantic dimensions of the negative emotion domain and increased use of the distal demonstrative for dimensions of the positive emotion domain, compared to control subjects. This may reflect an increased psychological proximity of negative emotion domains accompanied by increased psychological distance of the positive emotion domains"* (line 459-464).

Reviewer 2: Why are determiners only added for German and Tagalog (table 2)?

To clarify this, we have now added a short paragraph in the main text explaining that this is necessary, due to the fact that the spatial demonstratives in these two languages are *adverbs,* rather than adjectives, and hence requires determiners in grammatical coherence with the

noun gender (line 160-163). This section additionally refers to the S1 Experimental Procedures, in which the difference in demonstrative systems between languages and how they are implemented in the DCT paradigm are explained in detail. These two edits should hopefully address questions about differences in these translations.

Reviewer 2: The results for Chinese were apparently worse than the baselines - any idea why? Do you attribute this only to the different language families? I think there might also be a cultural effect (also for languages within Europe of course) which might be worth exploring.

Regarding the comment made by Reviewer 2 regarding the potential reasons for the poor Chinese performance, we agree that there might be cultural differences too. To fully highlight different potential explanations (in addition to language family) we have now edited the discussion section (line 563-581), which discusses how the semantic effects in the Chinese results do seem to resemble those of the other languages, however with much larger uncertainty and less robustness in the estimated effects. This could indicate that the most likely reason or the current differences are due to the (much) smaller sample size for Chinese, However, we agree that there may be cultural effects playing a role here as well, and we have included in the discussion the possibility that the tabood nature of depression in China could play a role (in different ways). Further, we have added a short paragraph (line 581-586) stating that this could also reflect two different kinds of cultural differences in the semantics and the use of demonstratives. However, any conclusions on this based on the present results are premature given the large difference in sample size and the low robustness of the estimates in the Chinese analysis, which is also clarified in this section. We thank you for the suggestion to more clearly and thoroughly highlight and discuss different potential explanations for these results, and believe that the paper is now more transparent and comprehensive in the treatment of these results and their interpretation.

Reviewer 1: The translation process of the English DCT seems still ambiguous. Did the authors ask professional human translators? Was machine translation involved? Did the authors manually refer to dictionaries on their own? Though the acknowledgements section mentions a potential translator for Chinese and Filipino, what the "help" exactly means is unclear. In addition to the current elaboration of different demonstrative systems, the detailed translation process should be explained in the S1 Supplementary

Experimental Procedures. This process affects the quality of DCT translations and, ultimately, the discussions and interpretations of the results.

Reviewer 1 made the note that the translation process of the paradigm itself was rather ambiguous and unclear, on which we fully agree. To accommodate this, S1 Experimental Procedures now includes a detailed section outlining the translation process, including who conducted the translations and how it was done, as well as measures taken to align translations across languages as much as possible. This is indeed a highly important methodological aspect of the paper and we are grateful that this was pointed out.

3 | Response to direct questions

Reviewer 2: Did you perform hyperparameter optimization on the NNs?

Yes, we did. We tested both a 2-layer and 3-layer NN with fixed amounts of units and fixed activation functions in each layer and hyperparameter optimization on *batch size* and *n epochs* hyperparameters. The details of this are reported in the section "Classification Model Comparison" (line 275-276). We agree, that for the analyses on individual languages the sample is most likely too small for an NN to learn any meaningful features. However, since the analysis using the full cross-linguistic sample had a rather large sample size (with >3800 subjects), there was reason to at least test whether the NN could better detect patterns than other models. In all cases, however, we found that the NN was overfitting greatly to the training set and to an extent that resulted in chance performance on test sets.

Reviewer 2: The imbalance of language-specific data samples and the apparent instability of the models (which might also be due to the data imbalance) introduce confounding factors that are difficult to ignore. I think you did all you could to mitigate these issues, but I am wondering if a much larger dataset size would give very different results.

This is a good point, and one we also attempted to treat adequately in the paper. Specifically, the bootstrapped estimates should give a good hint towards what results would look like with larger samples (as these represent averages across different subsamples of the data, i.e., introducing random variations). That is, the most important semantic features in the model (strongest predictors of depression) occurring in the bootstrap results indicate the features that are most robustly the strongest predictors across different subsets of the data (in which case features that appeared as highly important in just one subset will be averaged out, and thus the bootstrapped results should not include features that appeared strongly associated with

depression by chance (due to something particular in one small dataset)). Further, the fact that the cross-linguistic model generalizes well to these individual language samples (when predicting on hold-out test sets), also indicate that the strongest predictive effects seem to generalize well to these languages as well. However, the main differences in each of the smaller language samples and the nuances in these differences are hard to draw inferences from in the current case, given the low robustness of most of the features in these models. Indeed, these bootstrapped results provide indications on this question, however, they are merely indications, and we can only speculate what it would actually look like with larger samples (this is mainly an issue for the Chinese, Tagalog Filipino and Russian models, which have significantly less data than the remaining languages. Italian, Spanish and German results appear robust and so could suggest that at least 300-400 subjects are needed to get robust and reliable estimates across all semantic dimensions). However, it should also be noted that even in the English sample, individual variation across and within participants is large, while average effects across trials and participants are robust (e.g. see Kruse et al. (2024) in which similar results are obtained in different subsamples and across different studies). The DCT task thus entails a lot of variability, which is also to be expected from a (seemingly random) two-alternative forced choice task, but what the results of this paper and the previous paper indicate is that when averaging across many trials, semantic tendencies and effects begin to appear. This is also what we see in the present paper for German, Italian and Spanish, which exhibit similar robustness and results as the much larger English sample. These points, as well as the motivation for doing the robustness tests, are discussed in detail throughout the paper (lines 305-319, 376-388, 399-409, 445-478, 614-627).

Lastly, we again wish to thank the reviewers. Their comments and suggestions were highly constructive, sound and detailed, highlighting aspects of the paper that needed clarification or further explanation, as well as potential alternative points to be considered or discussed. The paper has now been revised to accommodate and integrate these comments as well as possible, aiming to reduce any confusion, questions or miscomprehension in the paper and provide more clarity on important aspects of the methods and conclusions.

On behalf of the authors,
Line Kruse
